# Disease-related p63 DBD mutations impair DNA binding by distinct mechanisms and varying degree

**DOI:** 10.1038/s41419-023-05796-y

**Published:** 2023-04-18

**Authors:** Christian Osterburg, Marco Ferniani, Dario Antonini, Ann-Sophie Frombach, Ludovica D’Auria, Susanne Osterburg, Rebecca Lotz, Frank Löhr, Sebastian Kehrloesser, Huiqing Zhou, Caterina Missero, Volker Dötsch

**Affiliations:** 1 Institute of Biophysical Chemistry and Center for Biomolecular Magnetic Resonance, Goethe University, 60438 Frankfurt, Germany; 2 CEINGE Biotecnologie Avanzate Franco Salvatore, 80145 Naples, Italy; 3 Department of Biology, University of Naples Federico II, 80126 Naples, Italy; 4 Departments of Human Genetics, Radboud Institute of Molecular Life Sciences, Radboud University Nijmegen Medical Centre, Nijmegen, Netherlands; 5 Departments of Molecular Developmental Biology, Faculty of Science, Radboud University, Nijmegen, Netherlands

**Keywords:** Biophysical chemistry, Diseases

## Abstract

The transcription factor p63 shares a high sequence identity with the tumour suppressor p53 which manifests itself in high structural similarity and preference for DNA sequences. Mutations in the DNA binding domain (DBD) of p53 have been studied in great detail, enabling a general mechanism-based classification. In this study we provide a detailed investigation of all currently known mutations in the p63 DBD, which are associated with developmental syndromes, by measuring their impact on transcriptional activity, DNA binding affinity, zinc binding capacity and thermodynamic stability. Some of the mutations we have further characterized with respect to their ability to convert human dermal fibroblasts into induced keratinocytes. Here we propose a classification of the p63 DBD mutations based on the four different mechanisms of DNA binding impairment which we identified: direct DNA contact, zinc finger region, H2 region, and dimer interface mutations. The data also demonstrate that, in contrast to p53 cancer mutations, no p63 mutation induces global unfolding and subsequent aggregation of the domain. The dimer interface mutations that affect the DNA binding affinity by disturbing the interaction between the individual DBDs retain partial DNA binding capacity which correlates with a milder patient phenotype.

## Introduction

**Fig. 1:**
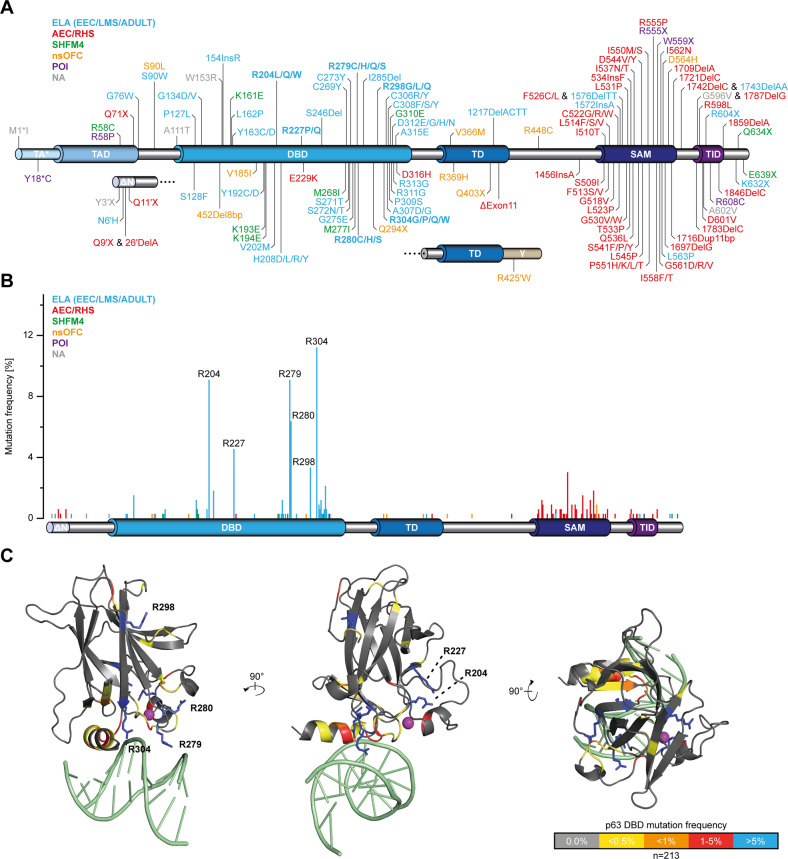
Analysis of reported disease-related p63 mutations. All published case reports of human developmental diseases linked to p63 mutations were extracted and evaluated (Supplementary Table S1). **A** An overview of the p63 mutation spectrum was generated by plotting position of all mutations on schematic domain architecture of p63. Each mutation is colour coded according to the predominantly caused developmental disease. **B** The extracted data was analysed for frequency of mis- and nonsense mutation occurring for each residue in p63 (*n* = 332 families). Frameshift mutations and gene deletions were disregarded due to their rare incidence. Mutation frequencies are plotted as bars along the p63 protein sequence aligned to the schematic domain architecture. Each bar is colour coded corresponding to the developmental disease the mutation of the respective residue is predominantly causing. The positions of the six mutational hotspots R204, R227, R279, R280, R298 and R304 are highlighted with labels. **C** Mutation frequency was calculated for the p63 DBD individually (*n* = 202 families) and blotted with the indicated colour code on the DBD structure (PDB: 3QYN). The six mutational hotspots are depicted as sticks and highlighted with labels. DNA is shown in light green and the zinc ion as a purple sphere. **A**, **B** TA*: TA*p63 isoform-specific N-terminus; ΔN: ΔNp63 isoform-specific N-terminus; TAD Transactivation domain; DBD DNA binding domain; TD tetramerization domain; SAM sterile-α-motif domain; TID transcriptional inhibitory domain; γ: p63γ isoform-specific C-terminus. nsOFC non‐syndromic orofacial clefting; POI premature ovarian insufficiency; NA not assigned to any of the known mutant p63-related diseases.

The transcription factor p63 is the master regulator of epithelial commitment, maintenance, and differentiation. Mouse studies have revealed that it is indispensable for epithelial, craniofacial and limb development with knock-out mice suffering from limb truncations and the lack of a multi-layered skin as well as of other epithelial structures [1, 2]. In contrast to the tumour suppressor p53, p63 is rarely mutated in cancer [3]. In accordance with its function in the development of epithelial structures heterozygous dominant‐negative p63 mutations are associated with distinct developmental disorders in humans [4–6] that feature at least one of three phenotypical hallmarks: ectodermal dysplasia (ED), limb defects, and orofacial clefting (OFC). The ectrodactyly, ectodermal dysplasia, and cleft lip/palate (EEC) syndrome (OMIM: 604292) presents the prototype of mutant p63 disorders as the patients exhibit all three hallmarks. Due to the high variability and overlap of the clinical phenotype, the EEC syndrome has been merged with two other syndromes, limb‐mammary‐syndrome (LMS) (OMIM: 603543; very similar to EEC with additional nipple and/or mammary gland hypoplasia) and acro-dermato–ungual–lacrimal–tooth (ADULT) syndrome (OMIM: 103285; similar to LMS but lacking OFC) into one clinical entity, the ELA (EEC, LMS, ADULT) syndrome. ELA mutations mostly cluster in the DNA binding domain (DBD) (Fig. 1A+B; Supplementary Table S1). Likewise, two other p63-related syndromes, the ankyloblepharon‐ectodermal defects‐cleft lip/palate (AEC) syndrome (OMIM: 106260) and Rapp‐Hodgkin syndrome (RHS; OMIM: 129400), can be combined to one entity, AEC/RHS with mutations residing in the α-C-terminus [7, 8]. AEC/RHS patients exhibit no limb defects but suffer from skin erosions (more severe in AEC than in RHS) and ankyloblepharon (only AEC) instead [9]. Mutations comprise mainly missense mutations in the sterile-α-motif (SAM) domain [10] but also missense mutations in the transcriptional inhibitory domain (TID) [11] and frameshift mutations in the α-C-terminus. In a previous study we could reveal the disease mechanism and show that AEC/RHS mutations either lead to the destabilization of the SAM domain with a concomitant exposure of two aggregation prone peptide sequences or create new aggregation prone peptides [12]. The loss of p63’s function is a direct cause of this mutation-driven aggregation. Finally, the non-syndromic disorder split hand/feet malformation 4 (SHFM4; OMIM: 605289) has no well-defined genetic basis as mutations are found throughout the p63 gene [13].

Contrary to the clear molecular mechanism (aggregation) that causes the AEC/RHS syndrome, the underlying molecular mechanism for the ELA syndrome and in particular the clinical variability is less well understood. For p53 which exhibits high sequence identity and structural homology a detailed understanding of the effects of mutations exists (Supplementary Fig. S1A+B; Supplementary Table S2). All p53 DBD mutations impair DNA binding and can be classified into five different groups based on their mechanism [14–17]: (1) DNA contact mutations target residues directly binding DNA, but do not affect the fold and stability of the DBD. In contrast, structural mutations suppress DNA binding by disturbing the fold, accompanied by a reduced thermodynamic stability of the DBD. They are further subdivided into mutations that either directly (2) or indirectly (3) disturb the zinc finger which is essential for the fold of the DNA binding interface. Other structural mutations locally distort the DNA binding interface without affecting the zinc finger (4) while mutations in the core of the DBD cause global unfolding of the metastable DBD and thereby impair DNA binding (5). These core mutations are also referred to as temperature sensitive mutations because DNA binding is retained at low temperatures, which do not induce global unfolding. In contrast to p53 and p63, to date neither cancer nor other human diseases have been linked to mutations in the p73 gene [3, 18, 19].

The high similarity between the p53 and p63 DBDs suggests similar molecular mechanisms and implies that the p53 mutation classification can be adopted for ELA mutations [20]. However, the spectra of p53 and p63 mutations do not completely overlap. Also, these domains differ in DNA binding cooperativity [21] and thermodynamic stability [22] (Supplementary Fig. S1C). To obtain a better understanding for the correlation of ELA syndrome characteristics and their underlying molecular impairment of the p63 function we have analysed the so far known p63 mutations in the DBD in detail and investigated their effect on transcription, DNA binding and stability. In addition, we have analysed the influence of selected mutations on the conversion of dermal fibroblasts into keratinocyte-like cells by RNA-seq, ATAC-seq and ChIP-seq analysis. Therefore, we focus here only on p63 mutations and compare them to p53. The high similarity between p63 and p73, however, make it likely that the results obtained here for p63 are directly relevant for p73 as well.

## Results

### DNA binding of p63 DBD mutants is impaired to varying degrees

Most DBD mutations that cause the ELA syndrome belong to six mutational hotspots (Fig. 1B, Supplementary Fig. S1D). R204, R227, R279, R280 and R304 account for most cases of EEC syndrome, while R298 is the ADULT syndrome hotspot. When mapped on the structure of the p63 DBD, these mutations primarily cluster in the DNA binding interface with only R298 located in the β‐sandwich fold with no connection to the DNA binding surface (Fig. 1C). Considering all known mutations, only few occur in the β‐sandwich part and are either located in loops or are surface exposed. Virtually all p63 mutations have counterparts in p53, but not always with a relevant frequency including the hotspots R227 and R298 (Supplementary Fig. S1E-G; Supplementary Table S2). The corresponding p53 residues R196 and R267 are either rarely mutated (R267) or only nonsense, but no missense mutations are known (R196). Inversely, p63 residues homologous to residues in the hydrophobic core of p53, including the p53 hotspots G245 and Y220, are not mutated at all. The only exception is R313 (R282 in p53), which is classified as a temperature‐sensitive mutation. However, R313 is found mutated exclusively to glycine, which is known to destabilize helices. These observations correlate with the high thermodynamic stability of p63 in comparison to the metastable p53 DBD [22].

**Fig. 2:**
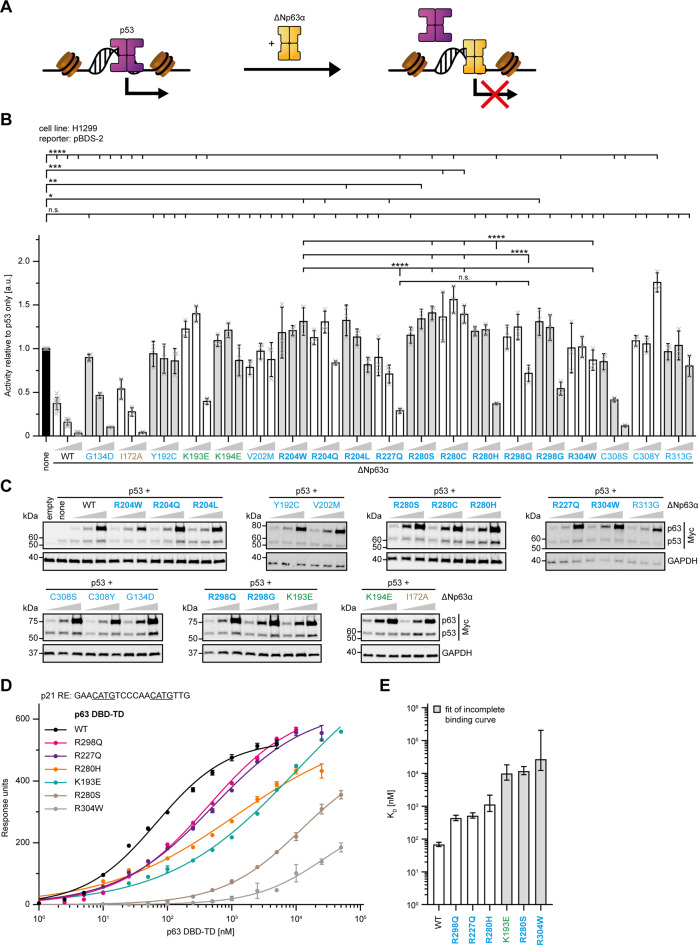
All DBD mutations negatively impact the DNA binding ability of p63. **A** Schematic representation of the principle of the luciferase reporter displacement assay used in the following study to assess DNA binding activity of p63 in cells. p53 (purple) but not ΔNp63α (yellow) is highly active on the used luciferase-based reporter. Addition of increasing amounts of p63 displaces p53 off the shared p53 family binding sites in the reporter resulting in a loss of luciferase transcription. Consequently, the measured luciferase signal is inversely correlated with the ability of p63 to compete with p53 for DNA binding. **B** Luciferase reporter displacement assay of ΔNp63α and the indicated DBD mutants using the pBDS-2 reporter. H1299 cells were transiently transfected with the respective luciferase reporter plasmids and either empty vector control, Myc-tagged p53 alone or in combination with increasing amounts of Myc-tagged p63 WT or mutants. The bar diagram shows the mean activity relative to p53 only and error bars the corresponding SD (*n* = 12 for p53 only and p63 WT, *n* = 3 for p63 mutants). Statistical significance was assessed by ordinary one-way ANOVA followed by Tukey’s post hoc test (Supplementary Table S3). n.s. *P* > 0.05, *P ≤ 0.05, **P ≤ 0.01, ***P ≤ 0.001, ****P ≤ 0.0001. **C** Expression levels of the transiently transfected proteins in the luciferase reporter displacement assay from (**B**) were determined by WB using an α-Myc antibody (Supplementary Fig. S7). GAPDH served as a loading control. **D** SPR affinity curves of purified p63 DBD-TD WT and indicated mutants binding to a 20 bp RE of the p21 promoter immobilized on a streptavidin (SA) chip. Measurements were repeated three times on the same chip. Data points were extracted by equilibrium analysis of sensograms (Supplementary Fig. S2K) and plotted with the error bars showing the corresponding SD. Binding curves were fitted with a non-linear, least squares regression using a single-exponential one-site binding model with Hill slope (Supplementary Table S3). **E** DNA binding affinities of purified p63 DBD-TD WT and indicated mutants derived from the fitted binding curves (**D**). The bar diagrams shows the equilibrium dissociation constants (K_D_) with the error bars corresponding to the 95% confidence interval. The bars are coloured in grey whenever an incomplete binding curve was fitted. **B**, **C**, **E** Mutations related to ELA syndrome are coloured in blue, mutations related to SHFM4 in green and artificial mutants in brown. Hotspot mutations are highlighted in bold.

To assess the impact of DBD mutations on the transcriptional activity of ΔNp63α we performed a ‘luciferase reporter displacement assay’ to measure semi‐quantitatively the DNA binding of ΔNp63α variants in the cellular environment (Fig. 2A). If there was no disease‐related mutation for a p63 residue, artificial mutations were designed based on the respective p53 cancer mutations (Supplementary Table S2). Cells were co‐transfected with reporter plasmids and p53 alone or in combination with increasing amounts of ΔNp63α variants. p53 is highly active on the chosen reporter, while the contribution of this p63 isoform is negligible. ΔNp63α cannot hetero‐oligomerize with p53 [23, 24] but is known to suppress its activity by displacing it off the shared response elements (REs) [25]. Thus, the inhibition of p53 by p63 only depends on its ability to bind DNA and the measured luciferase signal is inversely correlated with the degree of p53 displacement. Such a displacement assay provides more realistic information about the DNA binding behaviour of these mutant proteins than a classical luciferase-based transactivation assays (that we also performed, see Supplementary Figs. S2A-H, S7). The transient overexpression in these assays makes them susceptible to saturation effects and so partially active mutants can appear to be wild‐type‐like.

In the displacement assay, as expected, ΔNp63α wild‐type (WT) efficiently suppressed p53 activity with increasing concentration (Fig. 2B+C; Supplementary Fig. S7). In contrast, selected ELA mutations (Y192C, V202M, R204W, R304W and R313G) did not affect p53’s activity significantly. The effect of a mutation does not only depend on the site of the mutation but also on the type of amino acid present in the mutant: differential effects can be seen for example for the hotspot, R280: while mutation to serine or cysteine result in DNA binding incompetent protein, the histidine mutation shows residual binding. These results underscore the importance to analyse the exact mutant in each case. Surprisingly, the EEC hotspot mutation R227Q was able to displace p53 in a concentration-dependent manner. Interestingly, R227Q was shown to cause a less severe phenotype in patients compared to other EEC syndrome hotspot mutations [26]. This displacement assay also revealed that ADULT and SHFM4 mutations negatively affected DNA binding, despite their seemingly full activity in the luciferase reporter-based transactivation assay. The ADULT R298Q and R298G mutations as well as the SHFM4 K193E mutation led to a moderate reduction in DNA binding affinity, comparable to R227Q, while SHFM4 K194E was not able to displace p53 at all (Fig. 2B+C; Supplementary Figs. S2E+F, S7).

To assess the effect of ELA mutations on the DNA binding affinity directly in vitro, we used a DNA pull‐down assay with the REs of the K14 and p21 promoters as baits (Supplementary Figs. S2I+J, S7). In agreement with the displacement assay, R227Q and the artificial core mutations did not significantly affect the interaction of ΔNp63α with DNA while DNA binding was reduced to background levels for all other EEC syndrome mutants tested. Finally, we measured the DNA binding affinity of p63 WT and selected mutants by surface plasmon resonance (SPR) using recombinantly expressed and purified DBDs, either fused with the tetramerization domain (TD) or isolated, and 20 bp REs (Fig. 2D+E; Supplementary Fig. S2K-N). These data confirmed that R227Q, R298Q and R280H are still able to bind DNA although with a reduced affinity compared to p63 WT, while the affinities of R280S and R304W are further diminished. The SPR measurements also underscored that the SHFM4 mutation K193E reduces the DNA binding affinity.

Overall, the different types of measuring the DNA binding competence have shown that several ELA mutants still bind to DNA, albeit with a reduced affinity, that artificial core domain mutants behave like wild type and that SHFM4 mutations affect DNA binding as well. All results of the in vivo and in vitro DNA binding assays are summarized in Table 1.

**Table 1:** Summary of experimental data and classification of p63 DBD mutations.

Mutation	Syndrome	Position	Activity	DNA binding	Zinc loss	Destabilization	Classification
S272N	ELA	L3	−				DNA contact
R279H	ELA	L3	−		−	−	
R279C	ELA	L3	−				
R279Q	ELA	L3	−				
R304W	ELA	L3	−	−	+	−	
R304Q	ELA	L3	−		+	−	
R304P	ELA	L3	−		++	+	
C308S	ELA	S10/H2	+	+++			
C308Y	ELA	S10/H2	+	−			
A315E	ELA	H2	−				
Y192C	ELA	S4	+	+	++	+++	Zinc region
Y192D	ELA	S4	−				
K193E	SHFM4	S4/Ha	+	++	+	+	
V202M	ELA	L2A	+	+	++	+++	
R204W	ELA	L2A	−	−	+++	+++	
R204Q	ELA	L2A	+	+	++	+++	
R204L	ELA	L2A	+	+	++	+	
H208Y	ELA	H1	−		+++	+++	
C269Y	ELA	L3	−				
C273Y	ELA	L3	−				
R280S	ELA	L3	−	−	+	+	
R280C	ELA	L3	−	−	+	+++	
R280H	ELA	L3	+	++	+	+++	
154InsP	ELA	S2	+				H2 region
L162P	ELA	S2’	−				
Y163C	ELA	S2’	−				
C306Y	ELA	S10/H2	−				
C306R	ELA	S10/H2	−				
P309S	ELA	H2	−				
D312G	ELA	H2	−				
D312N	ELA	H2	−				
D312H	ELA	H2	−				
R313G	ELA	H2	−	−	+	+	
G134D	ELA	N-term.	+++	+++			Dimer interface
K194E	SHFM4	S4/Ha	+	−	+	−	
R227Q	ELA	S5	+	++	−	−	
R298Q	ELA	S10	+++	++	−	+	
R298G	ELA	S10	+++	++			

The table shows all p63 DBD mutations examined in this study with the associated and summarizes the most important experimentally determined parameters together with the resultant classification (see discussion). The position of the mutated residue is annotated in relation to the secondary structure elements of the DBD (H α-helix, S β-strand, L loop; see Supplementary Fig. S1A for details). The results of the luciferase reporter assays are displayed in the column ‘Activity’ (-: inactive; +: wildtype-like; +++: hyperactive). Data from the DNA pulldown assay, the luciferase reporter displacement assay and SPR measurements are integrated in the column ‘DNA binding’ (-: no DNA binding; +: residual DNA binding; ++: moderate DNA binding; +++: wildtype-like DNA binding). The tendency of the mutant DBDs to lose the zinc ion as a measurement for the destabilization of the zinc finger is stated in the column ‘Zinc loss’ (-: no zinc loss; +: minor zinc loss; ++: moderate zinc loss; +++: strong zinc loss). The destabilization of the mutant DBDs is displayed in the column ‘Destabilization’ (-: no destabilization or stabilization; +: moderate destabilization; +++: strong destabilization).

### Impact of mutations on zinc ion binding of the p63 DBD

In addition to directly disrupting the DNA binding interface, mutations in p53 have been identified that perturb binding of the zinc ion. Corresponding p63 mutations are found in ELA syndrome patients (e.g., H208Y and R204W, Supplementary Table S2). To study the structural consequences of removing the zinc ion from the p63 WT DBD the zinc-free apo form was investigated by NMR spectroscopy. Comparison of [^1^H‐^15^N]-BEST‐TROSY HSQC spectra of the ^15^N-labelled holo and apo forms showed a partial collapse of the chemical shift dispersion, suggesting a partial but not complete loss of structure (Supplementary Fig. S3A–C). Mapping of the chemical shift differences onto the structure of the DBD, revealed that the strong structural changes were limited to the zinc finger and its surrounding elements of the DNA binding interface like the loops L2 and L3 that contact the minor groove of the DNA, while the core of the domain remained unperturbed.

**Fig. 3:**
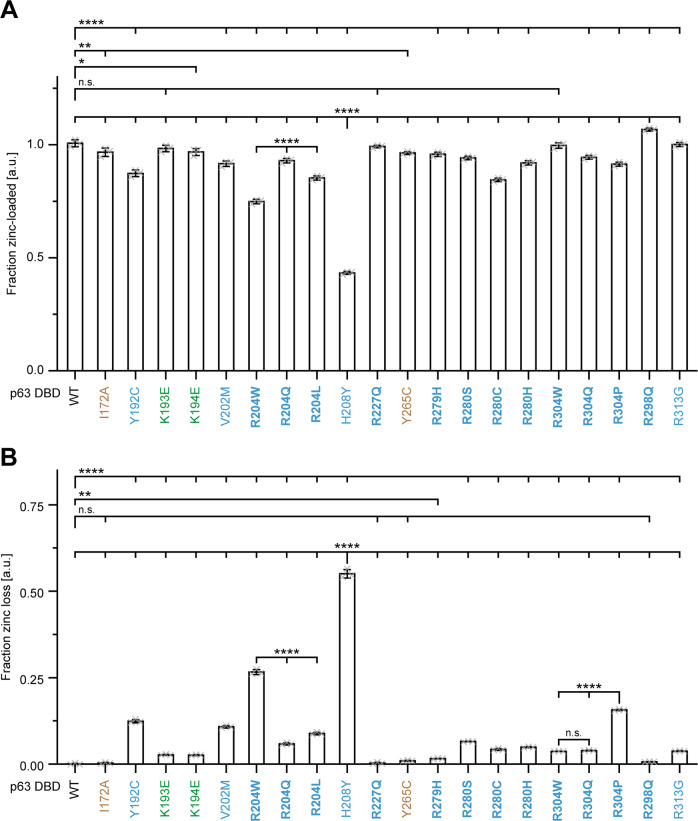
A subgroup of p63 DBD mutations destabilize the structurally important zinc finger. **A** Zinc content assay of the purified p63 WT and mutant DBDs. Bound zinc was released from the DBD zinc finger by an alkylation agent and the concentration was determined by a colorimetric analysis using the zinc binding dye PAR. The zinc concentration was referenced to the protein concentration. The bar diagram shows the mean fraction of DBDs in the holo form and error bars the corresponding SD (*n* = 3). **B** Zinc dissociation assay of the purified p63 WT and mutant DBDs. Release of the zinc ion from the zinc finger during incubation at 37 °C in presence of the PAR was measured via the specific absorbance of the PAR_2_-Zn^2+^ complex. In a second reaction the total zinc content was determined by complete release of the bound zinc with an alkylating agent. The DBD and PAR concentrations were 30 µM and 500 µM, respectively. The bar diagram shows the mean fraction of zinc dissociated in relation to the total zinc content and error bars the corresponding SD (*n* = 3). **A**, **B** Statistical significance was assessed by ordinary one-way ANOVA followed by Tukey’s post hoc test (Supplementary Table S3). n.s. *P* > 0.05, *P ≤ 0.05, **P ≤ 0.01, ***P ≤ 0.001, ****P ≤ 0.0001. Mutations related to ELA syndrome are coloured in blue, mutations related to SHFM4 in green and artificial mutants in brown. Hotspot mutations are highlighted in bold.

Next, we analysed the zinc loading of purified mutant DBDs. Most DBD mutants were purified in their holo form, but for H208Y and R204W ~50% and ~25% were in the apo form, respectively, while the Y192C, V202M, R204Q/L and R280C mutations caused a minor deficiency of zinc ions (Fig. 3A). To get further insight, the DBDs were incubated with the zinc complexing dye PAR which competes with the p63 DBD for zinc binding. As expected, PAR was not able to extract the zinc ion from the WT DBD. The same was true for DBDs carrying the ELA mutations R227Q or R298Q or the artificial core mutations I172A or Y265C. In contrast, H208Y and R204W showed a severe loss of zinc ions in the presence of PAR (Fig. 3B).

These data show that loss of Zn binding does not destabilize the DBD core but leads to rearrangement of structural elements important for DNA binding and can thus explain the impaired DNA binding triggered by Zn loss in the H208Y and R204W mutants. All results are summarized in Table 1.

### Thermodynamic destabilization of the p63 DBD by mutations

To assess the impact of zinc loss and mutations on the thermodynamic stability, the melting temperatures of purified DBDs were determined by thermal shift assay (TSA). As expected, the melting temperatures of the p63 DBD (57.2 °C) was significantly higher compared to the p53 DBD (40.5 °C). Removal of the zinc ion (29.9 °C) or introduction of the destabilizing mutation R175H (27.5 °C) reduced the melting point of the p53 DBD below physiological temperature. The apo form of the p63 DBD (51.0 °C) was destabilized to a similar extend but remained above the critical line of 37 °C (Supplementary Fig. S4A).

**Fig. 4:**
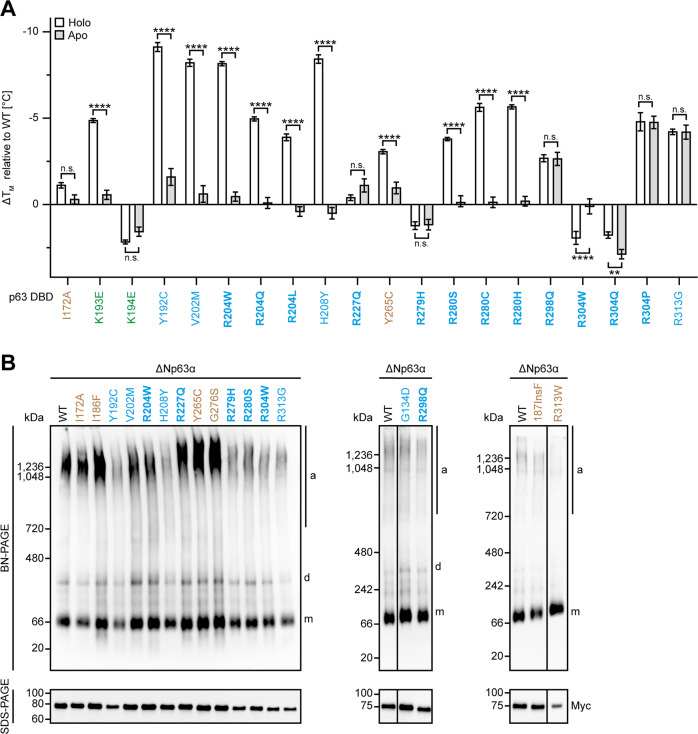
p63 DBD mutation affect the thermodynamic stability of the domain without inducing its aggregation. **A** Melting temperature difference (ΔTM) of mutant DBDs. The melting temperatures (Supplementary Fig. S4A) were referenced to the holo or apo form of the p63 WT DBD, respectively. The bar diagram shows the mean melting temperature and error bars the corresponding SD calculated by error propagation (*n* = 3). Statistical significance was assessed by ordinary one-way ANOVA followed by Tukey’s post hoc test (Supplementary Table S3). n.s. *P* > 0.05, *P ≤ 0.05, **P ≤ 0.01, ***P ≤ 0.001, ****P ≤ 0.0001. **B** BN-PAGE of ΔNp63α WT and indicated ELA and artificial DBD mutants. Cell lysates were subsequently analysed by BN-PAGE (upper panel) and SDS-PAGE (lower panel) followed by WB using α-Myc antibody (Supplementary Fig. S7). Oligomeric states are indicated by ‘m’ (monomer) and ‘d’ (dimer), while ‘a’ marks high molecular weight species corresponding to aggregates. **A**, **B** Mutations related to ELA syndrome are coloured in blue, mutations related to SHFM4 in green and artificial mutants in brown. Hotspot mutations are highlighted in bold.

Next, we analysed a set of mutant p63 DBDs. Neither the disease‐related nor the artificial core mutations decreased the melting point below 37 °C, not even in the apo form. R227Q did not alter the stability of the p63 DBD, while K194E, R279H and R304W/Q even slightly increased it. The latter is a known phenomenon for p53 mutations, which target surface exposed residues contacting the DNA (e.g., R248Q and R273H). All other mutations destabilized the p63 DBD, but to varying degrees (Fig. 4A). Accordingly, none of the tested mutations caused aggregation of p63 beyond the level seen in wild type p63 caused by the TID, which, however, can be suppressed by a V603D mutation [27]. In contrast destabilizing p53 mutations result in aggregation (Fig. 4B; Supplementary Figs. S4B+C, S7). Interestingly, mutations that are known to thermostabilize mutant p53 [28, 29] are able to reactivate exactly those p53 mutations that do not have a mutational counterpart in p63 (V143A, V157F, Y234C and R282W) (Supplementary Figs. S1C, S4D–F, S7). The absence of aggregation via the core DBD domain is not based on the absence of aggregation prone sequences. Like p53, the p63 DBD contains such sequences [27] that are usually hidden in the folded domain and only contribute to aggregation when exposed due to unfolding. This observation again shows that the core of the p63 DBD is thermodynamically more stable than the p53 core. The results are summarized in Table 1.

### A subset of p63 DBD mutants can induce trans-differentiation of human dermal fibroblasts into keratinocyte-like cells

Our results so far indicate that most mutations affecting the DNA binding interface directly or inducing a shift to the zinc-free apo state, strongly impair the affinity to DNA and concomitantly the transcriptional activity. The effect of some mutations, however, cannot be explained with this scheme. These are the EEC syndrome mutation R227Q, the ADULT syndrome mutation R298Q and the SHFM4 mutations K193E and K194E.

**Fig. 5:**
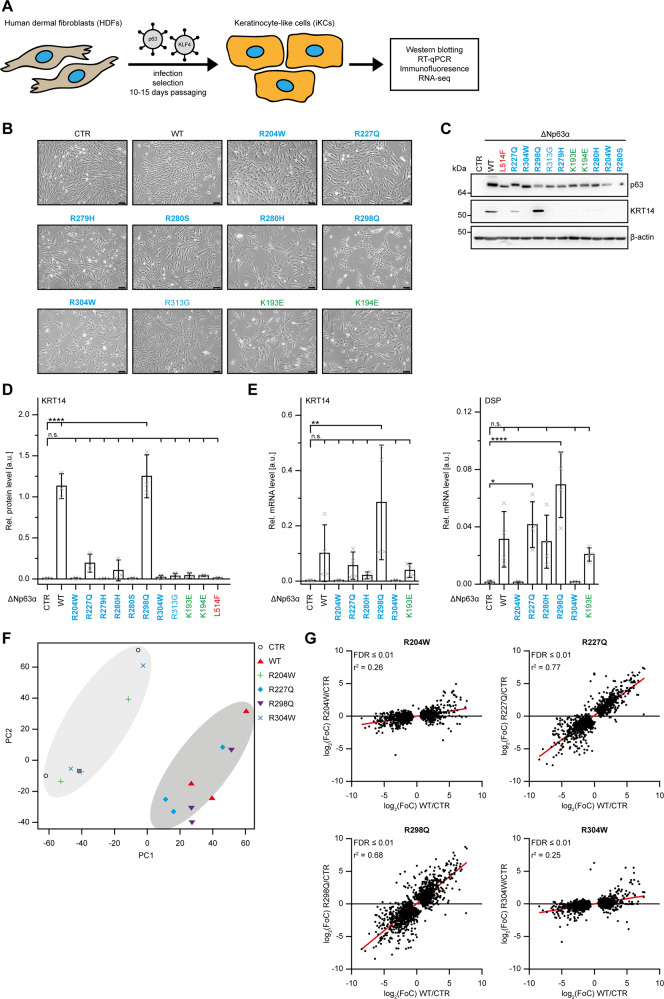
A subset of p63 mutants can induce trans-differentiation of human dermal fibroblast into keratinocytes-like cells. **A** Schematic representation of the experimental setup for conversion of human dermal fibroblasts (HDFs) to keratinocyte-like cells (iKCs). HDFs are retrovirally transduced with KLF4 only or in combination with ΔNp63α, selected and passaged for 10–15 days to achieve complete conversion. Cells were subsequently analysed by WB, RT-qPCR, immunofluorescence, and RNA-seq. **B** Cell morphology of HDFs transduced with either KLF4 alone (CTR), WTp63 or the indicated p63 DBD mutants at ten days post-infection. Scale bar (black), 50 µm. **C** Expression of p63, KRT14 and β-actin in HDFs transduced with KLF4 alone (CTR), WTp63 or the indicated p63 mutants 15 days post-infection was assessed by WB (Supplementary Fig. S7). **D** Quantification of KRT14 protein expression. The bar diagram shows the mean relative protein level and error bars the corresponding SD (*n* = 3). **E** mRNA levels of KRT14 and DSP in HDFs transduced with KLF4 alone (CTR), WTp63 or the indicated p63 mutants 15 days post-infection. The bar diagram shows the mean relative mRNA level and error bars the corresponding SD (*n* = 4, except *n* = 3 for R204W and R304W). **F** Principal Component Analysis (PCA) of the RNA-seq samples expressing KLF4 alone (CTR), WTp63 or the indicated p63 mutants. **G** Linear regression (red line) and Pearson correlation coefficient of the indicated p63 DBD mutants compared to WTp63 (FDR < 0.01) (Supplementary Table S3). **D**, **E** Statistical significance was assessed by ordinary one-way ANOVA followed by Tukey’s post hoc test (Supplementary Table S3). n.s. *P* > 0.05, *P ≤ 0.05, **P ≤ 0.01, ***P ≤ 0.001, ****P ≤ 0.0001. **B**–**E** Mutations related to ELA syndrome are coloured in blue, mutations related to SHFM4 in green and mutations related to AEC/RHS in red. Hotspot mutations are highlighted in bold.

To address the molecular and functional behaviour of these mutants in a more physiological context, we took advantage of the ability of ΔNp63α WT to convert human dermal fibroblasts (HDF) into keratinocyte-like cells (iKC) in the presence of the transcription factor KLF4 [12, 30] (Fig. 5A). HDF were transduced with KLF4 and ΔNp63α WT or mutants. After 10–15 days in culture, conversion was evaluated by a change in cell morphology (Fig. 5B) and expression of the p63 target genes keratin KRT14 and desmoplakin (DSP) [31, 32] (Fig. 5C-E; Supplementary Figs. S5A, S7). As expected ΔNp63α WT efficiently converted HDF into iKC, whereas most EEC mutants (R204W, R304W, R313G, R279H, R280S, R280H) and the AEC mutant L514F were unable to induce conversion. SHFM4 mutants (K193E and K194E) showed an intermediate ability to convert with a low number of KRT14 positive and more flattened and elongated cells. Interestingly, in partial agreement with their residual DNA binding affinity, the mutants R227Q and R298Q were able to convert as efficiently as ΔNp63α WT at least as judged by the morphological changes and KRT14 and DSP expression.

To further characterize the transcriptional consequence of selected mutations, we compared transcriptomic analyses of HDF cells transduced with KLF4 in combination with p63 WT or DBD mutants at the end of the conversion protocol. Principal Component Analysis (PCA) revealed that the two dead mutants R304W and R204W clustered with control samples lacking exogenous p63 as expected, whereas the R227Q and R298Q clustered more closely with p63 WT (Fig. 5F). Accordingly, analysis of differentially expressed genes showed no significant gene perturbation for the R304W and R204W mutant expressing cells as compared to control cells lacking p63, whereas R227Q and the R298Q mutants affected a relatively large number of genes with significant overlap with those affected by p63 WT (Fig. 5G; Supplementary Fig. S5B). These data indicate that cells expressing p63 R227Q or - to a lesser extent - p63 R298Q maintain most of the function of p63 WT, whereas R304W and R204W mutants are completely impaired.

### The mutants R227Q and R298Q bind to incomplete p63 consensus sequences

**Fig. 6:**
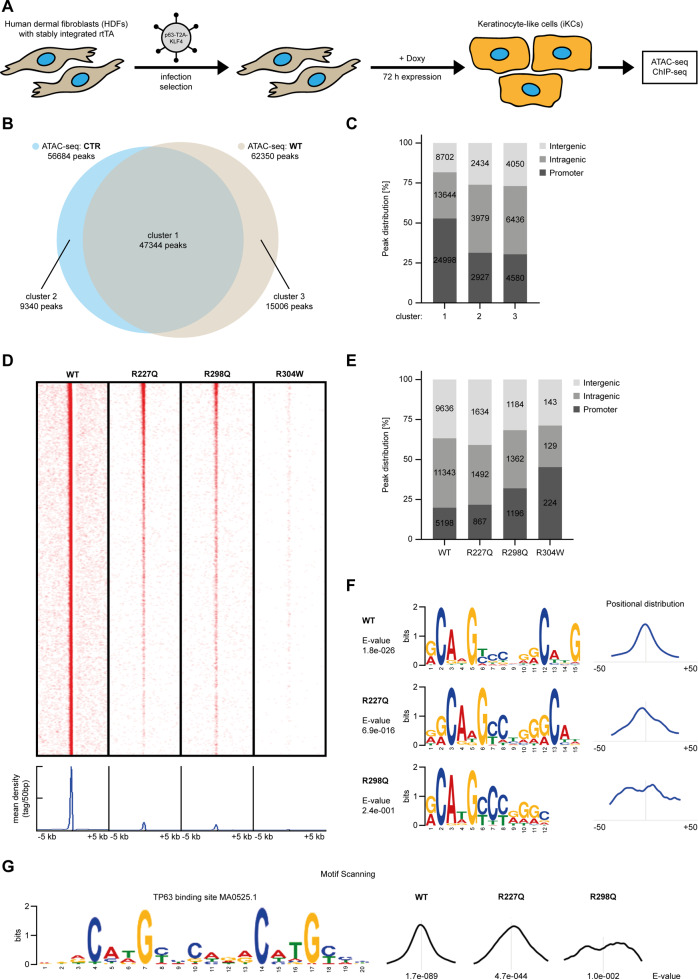
p63 R227Q and R298Q bind to incomplete consensus sequences. **A** Schematic representation of the inducible system for conversion of human dermal fibroblasts (HDFs) to keratinocyte-like cells (iKCs). HDFs with a stable integration of a reverse tetracycline-controlled transactivator (rtTA) are retrovirally transduced with KLF4 only or C-terminally fused to ΔNp63α with a self-cleaving T2A peptide. Conversion is achieved by doxycycline (Doxy)-induced expression of p63 and KLF4 for 72 h. Cells were subsequently analysed by ATAC- and RNA-seq. **B** Venn diagram of ATAC-seq peaks in HDF expressing either WTp63/KLF4 (light brown) or KLF4 alone (CTR; in light blue) showing the number of open regions (-Log10FDR ≥ 10). **C** Percentage of ATAC-seq peak distribution in promoters, intergenic, or intragenic regulatory elements of the sequences open in both CTR HDF and p63 expressing HDF (cluster 1), in those open only in CTR HDF (cluster 2), or in p63 expressing HDF (cluster 3). **D** Upper panel: Heat map representation of the signal density of p63 WT and the indicated DBD mutants sequence tags with a stringency of -Log10FDR ≥ 20. p63 binding sites were distributed within ±5 kb from the WT peak summit. Lower panel: Mean read densities centred (±5 kb) around the peak summits. **E** Percentage of peak distribution bound by p63 WT or the indicated DBD mutants in promoters, intergenic, or intragenic regulatory elements. **F** De novo discovery motifs using STREME performed on 100 bp spanning the summits of the top 1000 ChIP-seq peaks (-Log10FDR ≥ 20) of p63 WT and DBD mutants. E-value, and the distribution are shown. **G** p53 p63 consensus motif scanning (MA0106.30525.1 (TP53)) identified by CentriMo using MEME-ChIP. E-value, and the distribution are shown.

To determine whether the mutants R227Q and R298Q that retain the ability of inducing iKC commitment can bind chromatin to the same extent as ΔNp63α WT, we exploited a doxycycline inducible conversion system expressing either KLF4 alone, or KLF4 and p63 together in the presence of doxycycline [33] (Fig. 6A). This set up allows to detect early chromatin changes upon p63 expression (Supplementary Figs. S6A, S7). Firstly, to identify putative regulatory regions, chromatin accessibility was assessed by ATAC-seq in BJ-HDF expressing KLF4 alone or ΔNp63α WT and KLF4 72 h upon doxycycline treatment. Approximately 57000 genomic regions were in an open conformation in HDF expressing only KLF4, and ~62000 were in an open conformation in HDF expressing KLF4 and p63 (Fig. 6B). Comparison of the two conditions revealed that the majority of accessible regions were open independently of the presence of p63 (cluster 1), whereas a smaller fraction changed chromatin conformation in the presence of p63, being closed (cluster 2) or open (cluster 3) only in the presence of p63. Interestingly, regions affected by p63 expression were enriched in distal intergenic or intragenic regions, whereas those open independently of p63 were enriched in promoter regions (Fig. 6C), suggesting that the presence of p63 affected mainly chromatin conformation of the distal regions.

To determine the ability of WT and mutant p63 to associate with chromatin, ChIP-seq with anti-p63 antibodies was performed. ~26000 p63 binding sites were identified in cells expressing ΔNp63α WT (Fig. 6D+E). Consistent with previous reports [34–36] and with the ATAC-seq data, almost 80% of the p63 binding sites were located at non-promoter regions (Fig. 6E), indicating that p63 is primarily involved in gene regulation through distal cis-regulatory regions. Integration with ATAC-seq data revealed that 53% of ΔNp63α binding regions were in an open chromatin conformation, of which 50% were open only in the presence of p63 binding (Supplementary Fig. S6B).

Next, we analysed the ability of mutant p63 to associate with chromatin. The R227Q and R298Q mutants bound only to a subset of regulatory regions bound by ΔNp63α WT, and with a reduced signal intensity (Fig. 6D+E; Supplementary Fig. S6C). In addition, regions enriched in p63R298Q binding accumulated in promoters, rather than in distal regions (Fig. 6E). Interestingly, most p63R227Q and p63R298Q binding regions were enriched for regulatory regions that were in an open conformation (73% and 75% respectively), and that were in an open conformation independently of p63 binding (64% and 70% respectively) (Supplementary Fig. S6B). In contrast, the dead mutant R304W displayed a very low number of binding regions mostly located at the promoters (Fig. 6E) and such regions were in an open conformation (87%) and were open independently of p63 (92%) (Supplementary Fig. S6B).

To identify potential differences in the preferred DNA binding sequence, we performed de novo motif discovery on the top 1000 sequences bound by p63 WT, p63R227Q or p63R298Q. As expected, the most prevalent DNA motif identified in the DNA binding regions bound by p63 WT corresponded to a canonical p63 binding site located in the centre of most binding regions (90%) (Fig. 6F; Supplementary Fig. S6D). The second de novo motif corresponded to a canonical binding site for the KLF family of transcription factors (identified in 18.5% of the sequences) (Supplementary Fig. S6D), consistent with the ability of KLF4 to contribute to HDF to iKC conversion together with p63 [33]. The most prevalent DNA motif found in p63R227Q expressing cells corresponded to an incomplete p63 DNA binding site with a reduced enrichment (found in 68% of the sequences) and with a less sharp central distribution (Fig. 6F; Supplementary Fig. S6D). An even more dramatic divergence from the p63 WT behaviour was observed for p63R298Q, for which the most enriched motif corresponded to a KLF binding motif (60%), whereas a partial p63 consensus site was observed only in 22% of the sequences and was not centrally distributed. Similar results were obtained by performing motif scanning for the p63 consensus sequences and p63 hemi-sites (Fig. 6G; Supplementary Fig. S6E) indicating that consistent with the above observations, p63R298Q was less capable to bind the p63 full binding site. Analysis of p63 binding sites for well characterized p63 target genes revealed that most of the bona fide target genes for p63 have redundant p63 binding sites in their genomic region [33], some of which are bound by p63R227Q and p63R298Q, although to a lesser extent than p63 WT (Supplementary Fig. S6F).

## Discussion

**Fig. 7:**
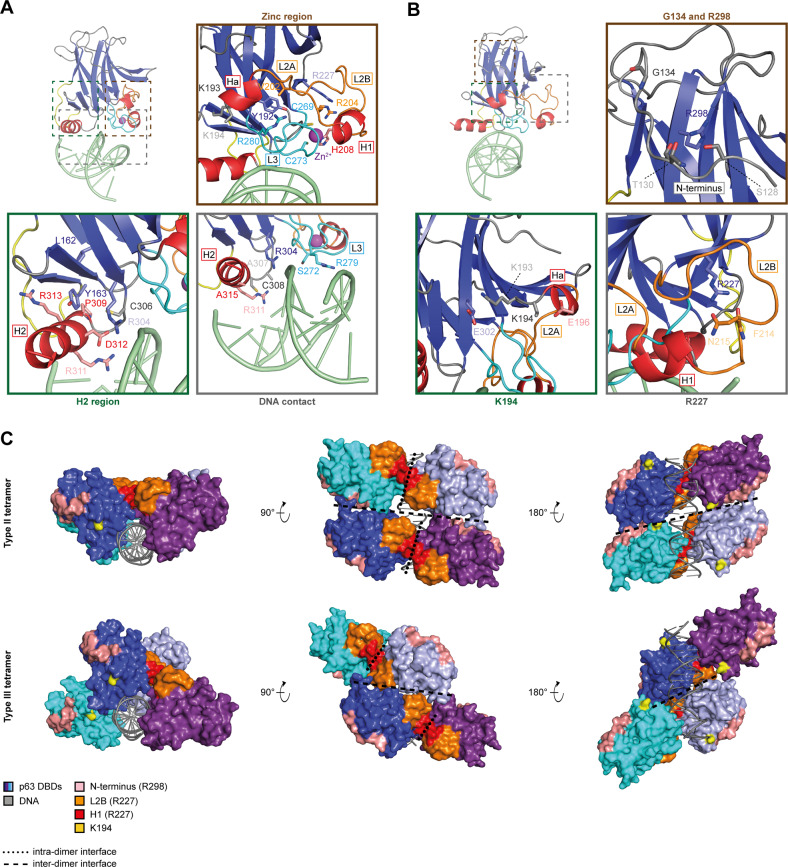
Classification of p63 DBD mutations based on their distinct mechanisms. **A** Most p63 DBD mutations belong to one of the three classes, which are based on the distinct mechanisms by which DNA binding is impaired: DNA contact (grey box), zinc region (brown box) and H2 region (green box). DNA contact mutations target residues contacting the DNA (S272, R279, R304, C308). An exemption is A315E, as A315 is not involved in DNA binding, but will lead to repulsion between the introduced glutamic acid and the negatively charged DNA backbone. Mutations of A307 and R311, the two remaining residues interacting with the DNA, have not been reported. Zinc region mutations affect DNA binding by perturbing the local fold of the zinc finger established by loops L2 (orange) and L3 (cyan) as well as helix H1 (red). The zinc finger positions the DNA contacting residue R279 and S272 binding the minor groove of the DNA. The mutations either target the zinc-coordinating residues (H208, C269 and C273) or adjacent, structurally important residues (Y192C, K193, V202, R204 and R280). R227 and K194 are located in this region as well. But R227 arranges L2B, the second part of loop L2 not involved in the structure of the zinc finger, and the side chain of K194 is surface exposed. H2 region mutations function by a similar mechanism but target the other half of the DNA binding interface composed of helix H2 (red) and the opposing β-strands S2, S2’ and S10 (blue) as well as loop L1 (yellow). This region positions the DNA contacting residues R304, C308 and R311 to bind the major groove of the DNA. Mutation of L162, Y163, C306, P309 or R313 perturb the local fold and thereby H2, while mutation of D312 directly alters the orientation of R304 and R311 (PDB: 3QYN). **B** Mutation of G134 and R298 (brown box), R227 (grey box) and K194 (green box) do not belong to the three major classes of p63 DBD mutations. The side chain of R298 aligns the N-terminus of the DBD via its interaction with S128 and T130. Loss of R298 or an introduction of an aspartate or valine at the position of G134 alters the conformation of the N-terminus. R227 contacts F214 and N215 and thereby arranges loop L2B, which is connected to helix H1. K193E most-likely perturbs the zinc finger by causing a charge repulsion with E302 which in turn affects helix Ha and loop L2A. The side-chain of K194, however, is surface exposed and not even fully resolved in this crystal structure due to high flexibility (PDB: 3QYN). **C** Four p63 DBD monomers bind to a full RE of 20 bp (grey) as a dimer of dimers (dark blue, purple and cyan, light blue) in two different conformations, named type II and type III. The two tetramer types assemble by distinct inter-dimer interfaces due to the different orientation of the dimers to each other (PDB: 3QYM). G134D and R298Q/G affect the N-terminus of the DBD (salmon), which is part of the inter-dimer interface in the type II tetramer. The side chain of the surface-exposed residue K194 (yellow) lies in the same interface. R227Q influences the conformation of loop L2B (orange), which contributes to the inter-dimer interface of type III. Helix H1 (red) adjacent to loop L2B contributes to the intra-dimer interface between two DBD monomers. **A**, **B** Structural elements are coloured according to Supplementary Fig. S1A and are labelled with boxes. DNA is shown in light green and the zinc ion as a purple sphere.

Based on the presented data and supported by structural analysis and comparison with the well‐studied p53 cancer mutations we propose the novel classification of p63 ELA and SHFM4 mutations based on four different mechanisms of impairing DNA binding: mutations in the direct DNA contact interface, in the zinc binding region, in the H2 region or in the dimer interface (Table 1; Fig. 7).

### Direct DNA contact

The DNA binding interface can be divided in two structural elements (Fig. 7A; Supplementary Fig. S1A+B). The zinc finger, encompassing loop L2 and L3 and helix H1, organizes the DNA contacting residues R279 and S272, which bind the minor groove of the DNA. Helix H2 together with the opposing β‐strands S2, S2’ and S10 and loop L1 form the part of the interface, which positions the residues contacting the major groove of the DNA. Mutations cluster in both sub‐structures. Interestingly, A307 and R311, the remaining two DNA contacting residues, have not been reported to be mutated in patients.

### Zinc binding region

Zinc region mutations act by perturbing the local fold of the zinc finger. They target either the zinc‐coordinating residues directly or adjacent structures essential for the local fold (Fig. 7A). They are characterized by exclusively destabilizing the holo form of the DBD and thereby favouring the zinc‐free state (Fig. 5A). Strikingly, the SHFM4 mutation K193E partially impairs DNA binding and presents all characteristics of a zinc region mutation (Table 1). K193 is mostly surface exposed, but introduction of a glutamic acid at this position most‐likely leads to charge repulsion with E302 located on the neighbouring β‐strand S10. The resulting conformational disturbance is transmitted to the zinc finger via helix Ha and loop L2A (Fig. 7B).

### H2 region

H2 region mutations cluster in and around helix H2 (Fig. 7A). They either perturb the positioning the DNA contact residues R304 and R311 directly or the complete local fold. Interestingly, all H2 mutations abolished DNA binding completely except for the ADULT mutation 154InsP (Table 1).

### Dimer interface

This class of mutations is rather small encompassing only five mutations: G134D, K194E, R227Q and R298Q/G. They impair DNA binding by varying degrees. The mutations do not disturb the zinc finger and are scattered across the DBD (Table 1 and Fig. 7B). R298 contacts S128 and T130 of the DBD N‐terminus, thereby contributing to its rigid conformation. Loss of the positively charged side chain disrupts the fold of the N‐terminus locally. Introduction of an aspartic acid or valine at the position of G134 has most‐likely a similar effect. R227 lies in the zinc region of the DNA binding interface, but with large distance to the coordinated zinc ion. It does not contribute to the structure of the zinc finger but to the conformation of the loop L2B by interacting with F214 and N215. R227Q thereby perturbs the L2B structure, and possibly the adjacent helix H1, without affecting the zinc finger.

The common putative mode of action of these mutations only becomes apparent when considering their position and local effect in a DNA‐bound tetramer. Crystal structures revealed that four DBDs assemble on the DNA as a dimer of dimers with two distinct conformations, type II and type III tetramers that interact via different inter‐dimer interfaces [37, 38] (Fig. 7C). Strikingly, the N‐terminus contributes to the inter‐dimer interface of the type II tetramer, while loop L2B is part of the type III inter‐dimer interface. In contrast, helix H1 is involved in the intra-dimer interface. The side chain of K194, which is surface exposed in the monomeric DBD, points towards the inter‐dimer interface of the type II tetramer. Accordingly, this class of mutations most‐likely impairs DNA binding by targeting the DBD‐DBD interfaces, which are necessary for high affinity binding of p63 to DNA [37, 38]. This effect on the inter-dimer interface is limited to canonical full sites comprised of two conjoined half‐sites without a spacer, because otherwise there is no interaction between the two dimers.

The classification described above is based on all currently known mutations. However, as ELA is a rare syndrome, the available number of patient data is relatively small in comparison to mutant p53 data collected from cancer patients. Consequently, the current absence of certain putative p63 mutations in the literature does not necessarily imply that they cannot occur in p63‐related diseases. Nevertheless, the absence of mutations in the hydrophobic core of the p63 DBD correlates with these mutations not affecting p63’s transcriptional activity in our luciferase-based assays. In contrast to p63, the corresponding p53 residues are cancer mutations that result in global unfolding of the p53 DBD.

Our analysis has revealed that several mutations in the dimer interface reduce the binding affinity without completely abrogating it. This category includes the SHFM4 mutations. So far, it was assumed that both K193E and K194E act by mutating putative ubiquitination sites in the DBD, just as the other SHFM4 mutations in the C‐terminus remove the sumoylation site [39–41]. Nevertheless, misregulation of p63 by blocking PTMs could further contribute to the pathogenic mechanism of K193E and K194E and explain their phenotypic association with SHFM4 instead of ELA as DBD mutations.

In addition to the SHFM4 mutations R227Q and R298Q behave differently from “classical” ELA syndrome mutations by retaining a relatively high DNA binding affinity and are capable of partially converting HDF into iKC. Interestingly, the patient phenotypes for the SHFM4 (only limb malformation), R227Q (mild EEC phenotype) and R298Q/G (no orofacial clefting) mutations is milder than for other ELA mutations that completely impair DNA binding, suggesting a correlation between residual DNA binding and severity of the phenotype. Of particular interest is the result that R298Q seems to bind only to hemi-sites which is consistent with a perturbation of the interdomain contacts within the mutated tetramer. Investigations of the spacer length between half sites on the binding affinity of p53 and p63 has revealed that p63 tolerates only a single nucleotide spacer while p53 is more flexible [38, 42]. Disturbing the DBD-DBD interface, seems to be a mechanism that has more effect on p63 while mutations in the core of the protein and a concomitant destabilization is a mechanism that exists only for p53.

The results described here are likely relevant for p73 as well, though no human disease caused by p73 mutations has been identified so far. While its thermostability is lower compared to the p63 DBD [43, 44], the p73 DBD is still significantly more stable than the p53 DBD which most likely prevents mutation-induced global unfolding as seen for p53. The high sequence identity with p63 further suggests that the four mechanisms that impair DNA binding of p63 will apply to p73 as well.

## Material and methods

### Mutation analysis

All published case reports of human developmental diseases related to p63 mutations were extracted from the PubMed database (as of September 2022) and analysed for mutation distribution and frequency (Supplementary Table 1). Whenever the number of patients or families carrying a mutation was not clearly stated, the minimal number was assumed for subsequent frequency calculations (recognizable by ‘≥’ in the table) resulting in a total number of 663 patients from 332 families. Each mutation was affiliated with the individual disease it predominantly causes.

While ΔNp63α is the relevant isoform for mutant p63-related developmental diseases (with very few exceptions of mutations in the N-terminus of TA*p63 and TAp63 (Fig. 1A)), the amino acids numbering applied in this manuscript is based on the TAp63 isoform, which is the standard in the literature. But as the numbering in reference to the TA*p63 and ΔNp63 isoforms is sporadically used as well and isoform-specific mutations exist, it was also included in the data analysis for clarity (Supplementary Table 1).

### Protein sequence and structure analysis

The protein sequences of the p53 and p63 DBD were aligned using the Clustal Omega web tool (Multiple Sequence Alignment, https://www.ebi.ac.uk/Tools/msa/clustalo/) [45, 46]. All protein structures were visualized, analysed and aligned using Pymol 1.5.0.3. (Schrödinger, Inc., USA).

### Molecular Cloning

For transient protein expression in mammalian cells or in-vitro translation of N-terminally Myc-tagged p53 and ΔNp63α, PCR-generated inserts were introduced in pcDNA3.Myc by subcloning using BamHI and XhoI restriction sites yielding pcDNA3.Myc-p53 and pcDNA3.Myc-ΔNp63α. pcDNA3.Myc is a derivative of pcDNA3.1(+) (Thermo Fisher Scientific) engineered with a Myc-tag between HindIII and BamHI sites. All gene inserts lack the intrinsic start codon to avoid expression of untagged proteins via alternative translation initiation skipping the Myc-tag. Any mutations were subsequently introduced by site-directed mutagenesis.

For recombinant expression of the p53 DBD (aa 94–291) and p63 DBD (aa 123–322) in *E. coli*, PCR-generated inserts were introduced in pET-15b (Novagen) by subcloning using NcoI (providing the start codon) and XhoI restriction sites, simultaneously adding a C-terminal tobacco etch virus (TEV) protease cleavage side followed by a His6-tag (AENLYFQGHHHHHH) via the 3’-oligo, yielding pET-15b-p53DBD-TEV-His6 and pET-15b-p63DBD-TEV-His6. For recombinant expression of the p63 DBD-TD (123–416), PCR-generated inserts were introduced in pET-15b-His10-TEV (N-terminal His10-tag followed by TEV protease cleavage site (MGHHHHHHHHHHDYDIPTTENLYFQGS), inserted via NcoI and BamHI) by subcloning using BamHI and XhoI restriction sites yielding pET-15b-His10-TEV-p63DBD-TD. For p63 an *E. coli* codon-optimized sequence of p63 was used, as previously described [47]. Any mutations were subsequently introduced by site-directed mutagenesis.

pBABE.Myc-ΔNp63α was created by introducing a PCR-generated insert of p63 in pBABE.Myc by subcloning using BamHI and XhoI restriction sites. pBABE.Myc is a derivative of pBABE-puro [48] engineered by exchanging the EcoRI site to a XhoI site, adding a new EcoRI site 5’ to the BamHI site and subsequently introducing a Myc-Tag between EcoRI and BamHI sites. The p63 gene insert lacks the intrinsic start codon to avoid expression of untagged proteins via alternative translation initiation skipping the Myc-tag. Any mutations were subsequently introduced by site-directed mutagenesis.

pRetrox-TRE3G-Myc-T2A-KLF4 was created as described in the following: An insert composing of a TRE3G promoter followed by a XbaI restriction site, a Myc-tag and BamHI site and NotI restriction sites was introduced into the pRetroX backbone (pRetroX GFP T2A Cre was a gift from Floris Foijer (Addgene plasmid #63704; http://n2t.net/addgene:63704)) by subcloning using BglII and EcoRI restriction sites. KLF4 (Isoform 2, aa 10–479) was amplified by PCR from the FU-tet-o-hKLF4 vector (FU-tet-o-hKLF4) was a gift from Konrad Hochedlinger (Addgene plasmid #19777; http://n2t.net/addgene:19777; RRID:Addgene_19777; [49]), engineering a self-cleaving T2A peptide (GEGRGSLLTCGDVEENPGPGSG) at the N-terminus via the 5’-oligo, and inserted into the backbone by subcloning using XbaI and NotI restriction sites yielding the final vector. Subsequently, PCR-generated inserts of ΔNp63α wildtype and mutants were introduced by subcloning using XbaI and BamHI restriction sites with the N-terminal Myc-tag added via the 5’-oligo resulting in pRetrox-TRE3G-Myc-ΔNp63α-T2A-KLF4 and the respective mutant p63 derivatives.

### Protein expression and purification

#### Expression

*E. coli* BL21(DE3) Rosetta cells (SGC Frankfurt) were transformed with the respective *E. coli* expression plasmids for protein production. Cells were grown at 37 °C in 2xYT medium supplemented with 100 µM zinc acetate (Carl Roth) to an optical density (OD) of ~0.8. Protein expression was induced with 1 mM IPTG (Carl Roth) for 16–18 h at 18 °C. For labelled expression, cells were grown in LB medium at 37 °C to an OD of ~1, harvested by centrifugation and resuspended in M9 minimal medium supplemented with 100 µM zinc acetate and the appropriate isotopic labelling reagents (1 g/l ^15^NH_4_Cl (Cambridge Isotope Laboratories Inc.) and 4 g/l glucose (Carl Roth) for ^15^N-labelling; 1 g/l ^15^NH_4_Cl and 2 g/l ^13^C-glucose (Cambridge Isotope Laboratories Inc.) for ^15^N/^13^C-labelling) to an OD of ~0.3. Cells were then grown at 37 °C to an OD of ~0.8 and protein expression was carried out as described above.

#### Purification

After expression cells were harvested by centrifugation and resuspended in IMAC A buffer (25 mM HEPES pH 7.0, 400 mM NaCl, 5% Glycerol, 20 mM β‐ME,10 μM zinc acetate and 25 mM imidazole) supplemented with DNAse (Sigma-Aldrich), RNAse (Sigma-Aldrich) and self-prepared protease inhibitor. Cells were lysed by sonification and the lysate was cleared by centrifugation at 4 °C. All proteins were subjected to an initial immobilized metal affinity chromatography (IMAC) purification step. The supernatant was loaded onto HiTrap IMAC Sepharose FF column (Cytiva) pre-equilibrated in IMAC A buffer, bound proteins were washed with 20 column volumes (CV) IMAC A buffer and eluted with 2 CV IMAC B buffer (IMAC A with 300 mM imidazole). The eluted proteins were supplemented with TEV protease (1:50 w/w; self-prepared) for cleavage of the His-tag and dialysed against IMAC A (DBDs) or IMAC 50 buffer (DBD-TDs; IMAC A buffer supplemented with 50 mM imidazole) over night (ON) at 4 °C. TEV protease, the cleaved tag and any uncleaved protein was removed by a subsequent reverse IMAC step using IMAC A or IMAC 50 buffer. DBD-TDs were further purified by ion-exchange (IEX) chromatography. The salt concentration was reduced below 100 mM by dilution with IEX A buffer (25 mM HEPES pH 7.0, 50 mM NaCl, 5% Glycerol, 20 mM β‐ME,10 μM zinc acetate) prior loading on HiTrap Heparin HP columns (Cytiva). Bound protein was eluted with a gradient from 50 mM to 1 M NaCl over 20 CV using IEX B buffer (IEX A with 1 M NaCl). In a final polishing step, all proteins were subjected to size exclusion chromatography (SEC) using either a Superdex 75 or Superdex 200 SEC column (Cytiva) equilibrated in assay buffer (25 mM HEPES pH 7.5, 150 mM NaCl, 0.5 mM TCEP) or NMR buffer (50 mM BIS-TRIS pH 6.8, 100 mM NaCl and 0.5 mM TCEP). Monodisperse peak fractions were pooled, concentrated by centrifugation (Amicon Ultra Centrifugal Filters, Merck KGaA) and snap frozen in liquid nitrogen for storage at −80 °C until usage.

#### Apo DBD

The protocol to prepare the apo form of the p53 and p63 DBD by removing the zinc ion was adapted from Butler et al [50]. Purified DBDs in assay or NMR buffer were treated with 1/33 volume 10% acetic acid and 1/100 volume EDTA (1 M, pH 8) for 5 min on ice and pH was raised again by addition of 1.5 volume HEPES (1 M, pH 7.5). The resulting apo DBDs were then transferred in fresh assay or NMR buffer with HiTrap desalting columns (Cytiva).

### Zinc assays

Zinc dissociation assay and zinc content assay were adapted from Butler et al [50] and performed in assay buffer and clear 96-well flat-bottom plates (Greiner Bio One) with a final volume of 200 µl. The specific absorption of the PAR_2_-Zn^2+^ complex at 520 nm was measured using Spark multimode microplate reader (Tecan).

To determine the total zinc content, 30 µM DBDs were incubated with 500 µM zinc binding dye 4-(2-pyridylazo)resorcinol (PAR) (Sigma-Aldrich) and 2.5 mM alkylating agent MMTS (Sigma-Aldrich), which completely and irreversible releases the zinc ions from cysteine zinc finger proteins like the DBDs, for 5 min at room temperature (RT). The fraction of DBDs in the zinc‐loaded holo form was determined by calculating the absolute zinc concentration with the help of ZnCl_2_-PAR standard curve and normalizing it to the protein concentration.

To measure the zinc dissociation, 30 µM DBDs were incubated with PAR and MMTS, as described above, and only in the presence of 500 µM PAR, which competes with the zinc finger for the zinc ion, for 15 min at 37 °C. The fraction of dissociated zinc was calculated by normalizing the dissociation sample with PAR only to the respective zinc content sample with PAR and MMTS.

Zinc assays were performed in three independent replicates from the same protein purification batch. Statistical significance was assessed by an unpaired *t*-test or ordinary one-way ANOVA followed by Tukey’s post hoc test using Graphpad Prism 8 (Supplementary Table 3).

### Thermal shift assay

Thermal shift assay (TSA) was performed using an iCycler iQ PCR Thermal Cycler (Bio‐Rad) and the SYPRO Orange fluorescent dye (Thermo Fisher Scientific) to determine melting temperatures. Prior measurement, purified DBDs were centrifuged at 16,000 xg and 4 °C for 15 min to remove aggregates. During all preparation steps, samples were kept on ice. Samples were prepared for measurement by mixing 36 μl protein working solution (35 µM in assay buffer) and 4 μl SYPRO Orange working solution (1:200 dilution in assay buffer) in MicroAmp Optical 96‐well plates (Thermo Fisher Scientific). For the measurement, a thermal gradient from 20 °C to 95 °C with a slope of 1 °C/min was applied and dye fluorescence was recorded every 0.2 °C. The negative first deviation of the fluorescence signal (‐dF/dT) was normalized and smoothed (using Graphpad Prism 8 (GraphPad Software)) before extracting the melting point.

Melting temperatures were determined by three independent measurements from the same protein purification batch. Statistical significance was assessed by ordinary one-way ANOVA followed by Tukey’s post hoc test using Graphpad Prism 8 (Supplementary Table 3).

### NMR

NMR samples were prepared by supplementing the p63 DBD in NMR buffer with 150 μM DSS and 5% D_2_O as well as protease inhibitor (Roche). All experiments were carried out at a sample temperature of 298 K on Bruker (Rheinstetten, Germany) Avance spectrometers with proton frequencies of 600 and 950 MHz, equipped with cryogenic ^1^H{^13^C/^15^N} triple-resonance probes. [^1^H-^15^N]-BEST-TROSY HSQC spectra of ^15^N-labelled p63 DBD (100 µM) in holo and apo form, respectively, and 2D [^1^H-^15^N]-BEST-TROSY HSQC as well as 3D [^1^H-^15^N]-BEST-TROSY HNCACB and HN(CO)CACB [51] spectra of ^15^N/^13^C-labelled p63 DBD holo (350 µM) were recorded. Spectra were assigned using Sparky 3.114 software (T. D. Goddard and D. G. Kneller, UCSF, USA). The backbone assignment of the holo DBD was performed using the HNCACB and HN(CO)CACB spectra supported by a published assignment [52]. The [^1^H-^15^N]-BEST-TROSY HSQC spectrum of the apo DBD was partially assigned based on the spectra of the holo form.

### SPR

Specific DNA binding of p63 DBD-TDs and DBDs was measured by surface plasmon resonance (SPR) spectroscopy using a Biacore X‐100 system (Cytiva). The protocol was adopted from Chen et al. [37] and has been performed as previously described for p53 [53].

Proteins were dialysed against DBD SPR buffer (10 mM HEPES pH 7.5, 150 mM NaCl, 200 μM TCEP and 0.005% Tween-20) or DBD-TD SPR buffer (25 mM HEPES pH 7.5, 200 mM NaCl, 500 μM TCEP and 0.005% Tween-20) and centrifuged to remove aggregates. For the measurement, protein solutions with different concentration were prepared by serial dilution (factor 2–2.5). The 10 µM stock solutions of biotinylated annealed dsDNA oligonucleotides (1:4 ratio forward and reverse, Supplementary table 4) were diluted in the respective SPR buffer to a final concentration of 10 nM for immobilisation.

After priming the Biacore X‐100 with SPR buffer, the SA chip (Cytiva) was docked and its surface conditioned by three 1 min injections of SPR activation solution (50 mM NaOH and 1 M NaCl) and three 30 s injections of SPR regeneration buffer (25 mM HEPES pH7.5, 500 mM NaCl, 0.05% SDS). The dsDNA with the 20 bp p21 RE (DBD-TD) or p63 CS (DBD) was immobilized in flow cell F2 and an equal amount of the dsDNA with the random sequence in flow cell F1. The immobilisation was performed sequentially for the individual flow cells with multiple short injections of the respective dsDNA solution. Unbound DNA was removed by two consecutive 15 s injections of SPR regeneration buffer. An amount of biotinylated dsDNA corresponding to ~100 response units (RU) for the DBD-TD measurements and ~200 RU for the DBD measurements was captured onto the surface of each flow cells, respectively.

All measurements were performed at 20 °C and in the multi‐cycle format with at least one start‐up cycle followed by nine measurement cycles with increasing concentrations of protein. Each cycle comprised a 3 min injection and association phase followed by a 2 min dissociation phase. Afterwards the surface was regenerated by one or two consecutive 15 s injections of SPR regeneration buffer and washed for 5 min with SPR buffer before starting the next cycle. Measurements were performed consecutively on the same chip, in case of DBD-TD in three replicates of independently prepared dilution series.

Sensograms were background corrected and affinity binding curves were extracted by equilibrium analysis with the BIAevaluation software (Cytiva). Affinity curves were plotted and fitted with a non‐linear, least squares regression using a single‐exponential one‐site binding model with Hill slope to determine the dissociation constant K_D_, the Bmax value and Hill slope factor h (Graphpad Prism 8) (Supplementary Table 3).

### Cell culture

The non-small cell lung cancer cell line H1299 (ATCC) was used because of its p53 deletion and non-detectable or low endogenous levels of p63 and p73, respectively, resulting in low background levels for the functional assays in this study. H1299 cells were cultured in RPMI 1640 medium (Thermo Fisher Scientific) supplemented with 10% Fetal Bovine Serum (FBS, Capricorn Scientific), 100 U/ml penicillin and 100 µg/ml streptomycin (Thermo Fisher Scientific). HEK293T cells (a gift from Gian-Paolo Dotto) were used for virus production and cultured in Dulbecco’s in Dulbecco Modified Eagle Medium (Sigma) supplemented with 10% FBS (#ECS5000L, Euroclone) and 2 mM Glutamine. Newborn human dermal fibroblasts (HDF, #C0045C, Thermo Fisher Scientific) were cultured in DMEM high glucose with the 10% FBS (#ECS5000L, Euroclone). BJ-HDF were cultured in DMEM-F12 (#ECM0090L, Euroclone) supplemented with either 10% FBS (#ECS5000L, Euroclone) or 10% tetracycline negative FBS (#ECS0182L, Euroclone). All cell lines were cultured at 37 °C and 5% CO_2_ and were routinely tested for mycoplasma contaminations.

H1299 cells were transfected using either the Effectene (Qiagen) or the Lipofectamin 2000 (Thermo Fisher Scientific) transfection reagent according to the manufacturers’ protocols.

### Retroviral infection and conversion assay

High titer retroviral production was obtained in HEK293T cells by transient transfection of pMXs-Klf4 (pMXs-Klf4 was a gift from Shinya Yamanaka (Addgene plasmid # 13370; http://n2t.net/addgene:13370; RRID:Addgene_13370) [54]), pBABE-Myc-ΔNp63α or the indicated DNA binding domain mutants together with the amphotropic viral envelope plasmid (pAmpho) using PEI (Thermo Fisher Scientific) in 1:4 DNA to PEI ratio. Cell supernatants containing the retroviruses were collected 48 and 72 h after transfection. HDF were infected twice at 30% confluence with retroviruses carrying p63 and KLF4 [30] in the presence of 8 μg/ml Polybrene (#H-9268, Sigma). Cells were passaged, selected with 2 μg/ml puromycin 48 h after the second infections, and grown for 15 days in the absence of puromycin. Cells from each round of conversion assay were subsequently used for analysis by WB, immunofluorescence, RT-qPCR and RNA-seq. Each biological replicate of these experiments corresponds to an independent round of infection and passaging.

To generate the inducible tetracycline system, the reverse tetracycline-controlled transactivator (rtTA) was introduced via lentivirus Lenti-CMV-rtTA3-Blast (pLenti CMV rtTA3 Blast (w756–1) was a gift from Eric Campeau (Addgene plasmid #26429; http://n2t.net/addgene:26429)) transfected with packaging plasmids into HEK293T cells. Viral suspension was filtered and then supplemented with polybrene (8 mg/ml), to infect BJ-HDF (ATCC). Cells were selected for 1 week with Blasticidin (Thermo Fisher Scientific) at 4 µg/ml. pRetrox-TRE3G-Myc-T2A-KLF4 or pRetrox-TRE3G-Myc-ΔNp63α-T2A-KLF4 wildtype or mutants were used generate retroviruses to infect the BJ-HDF rtTA cell in the presence of polybrene for 2 h at 37 °C and cells were selected using 2 µg/µl puromycin for approximately one week. For ATAC- and ChIP-seq analysis, protein expression in the generated polyclonal stable BJ-HDF cell lines was induced for 72 h using doxycycline (1 µg/ml) which was replenished every 24 h.

### SDS-PAGE and Western blotting

Proteins were separated by SDS-PAGE using either the XCell SureLock Mini‐Cell SDS‐PAGE system (Thermo Fisher Scientific) or Mini-PROTEAN Tetra Cell SDS‐PAGE system (Bio-Rad) in combination with NuPAGE 4–12%, Bis-Tris Mini Protein gels (Thermo Fisher Scientific) or 4–15% Mini-PROTEAN TGX Stain-Free Precast Protein gels (Bio-Rad), respectively, and subsequently transferred onto PVDF membranes using either the XCell II blot system (Thermo Fisher Scientific) in combination with Immobilon-P transfer membranes (Merck KGaA) or the Trans-Blot Turbo Transfer system (Bio-Rad) in combination with the Trans-Blot Turbo RTA Midi 0.45 µm LF PVDF Transfer Kit (Bio-Rad) according to the manufacturer’s recommendation. Membranes were blocked for 1 h at RT in blocking solution (5% skim milk powder (Sigma-Aldrich) in TBS-t (TBS supplemented with 0.05% (v/v) Tween 20)) and incubated with primary antibody diluted in blocking solution ON at 4 °C. Membranes were washed three times with TBS-t, incubated with the appropriate peroxidase-conjugated secondary antibody (Jackson ImmunoResearch) in blocking solution and wash three times again. Chemiluminescence was detected with Lumi-Imager F1 (Roche) using Amersham ECL Prime WB Detection Reagent (Cytiva).

For analysis of HDF conversion assay, proteins were separated by SDS-PAGE and blotted onto Immobilon-P transfer membranes (Merck KGaA). Membranes were blocked with PBS 0.2% Tween 20 with 5% nonfat dry milk and incubated with primary antibodies for 2 h at or ON at 4 °C. After washing three times with PBS 0.2% Tween 20, membranes were incubated for 1 h at RT by using the appropriate horseradish peroxidase-conjugated secondary antibody (Bio-Rad). Detection was performed by chemiluminescence (Clarity, Bio-Rad).

The following primary antibodies were used in this study: Myc (clone 4A6, # 05–724, Merck KGaA, 1:1000), GAPDH (clone 6C5, #MAB374, Merck KGaA, 1:10000), p63 (#124762, Abcam, 1:500), KRT14 (#905301, Biolegend, 1:5000) and β-actin (#sc-69879, Santa Cruz, 1:5000).

Densitometric analysis of western blots was performed using ImageJ (Version 1.51). For the KRT14 protein level of HDF conversion assays, KRT14 signal of each sample was normalized to β-actin. Statistical significance was assessed by ordinary one-way ANOVA followed by Tukey’s post hoc test using Graphpad Prism 8 (Supplementary Table 3).

### Blue Native-PAGE

Blue Native(BN)-PAGE analysis to assess the oligomeric state and aggregation of p53 and p63 variants was performed as described previously [27, 55]. H1299 cells were harvested 24 h after transfection and lysed for 30 min at RT in 100 µl BN-PAGE lysis buffer (25 mM TRIS-HCl pH 7.5, 150 mM NaCl, 20 mM CHAPS, 1 mM DTT and 2 mM MgCl_2_) supplemented with 1x protease inhibitor (Roche) and 1 µl benzonase (Merck). Sample were supplemented with 3x BN-PAGE (60% glycerol and 15 mM Coomassie Brilliant Blue G-250) and separated for 1 h at 150 V, followed by 1.5 h at 250 V, using the NativePAGE Bis-Tris Gel System with 3–12% Bis-Tris Mini Protein Gels (Thermo Fisher Scientific) at 4 °C. Subsequent immunoblotting and detection was performed using the XCell II blot system (Thermo Fisher Scientific) as described above with the exception that membranes were destained with methanol and fixed with 8% acetic acid before blocking. For parallel SDS-PAGE analysis, lysates were supplemented with 5x SDS-PAGE sample buffer (250 mM TRIS pH 8.0, 7.5% (w/v) SDS, 25% (v/v) glycerol, 0.025% (w/v) bromphenol blue sodium salt and 12.5% (v/v) β-ME), boiled and subjected to SDS-PAGE followed by immunoblotting as described above.

### Immunofluorescence

For immunofluorescence, cells were fixed in 4% PFA and permeabilized with 0.5% NP40 in PBS. Cells were blocked using 0.5% NP40 in PBS supplied with 5% goat serum and incubated with specific antibody for KRT14 (#905301, Biolegend, 1:1000). Images were acquired using a Leica DMi8 microscope (Leica Microsystems) in combination with the software platform LAS X (Leica Application Suite X, Leica Microsystems).

### Luciferase reporter assay

#### Conventional luciferase reporter assay

H1299 cells were transfected with pRL-CMV (Promega), pGL3 Basic with p21 promoter [39] or with K14 promoter (a gift from Prof. Dr. Karen Vousden (Francis Crick Institute, London, UK)), and pcDNA3.1(+) as an empty vector control or pcDNA3.Myc plasmids encoding the indicated Myc-tagged p53 or p63 variants. 24 h after transfection, cells were harvested and resuspended in fresh medium. Per sample, 45 μl cell suspension was transferred into four wells each of a 96-well plate to determine the luciferase signal in technical quadruplicates. To prepare input samples for western blot analysis, the residual cells were centrifuged, resuspended in 100 μl 2x SDS-PAGE sample buffer and boiled. The luciferase reporter assay was performed using the Dual-Glo luciferase assay system (#E2940, Promega) according to the manufacturers’ recommendation. Luminescence signals were measured using a Spark multimode or a GENios Pro microplate reader (Tecan). The ratio of the Firefly to Renilla luciferase signal was calculated for each technical replicate and the resulting mean of each sample was normalized to the empty vector control and p53 or p63 wildtype to yield the relative activity for each biological replicate.

#### Luciferase reporter displacement assay

H1299 cells were transfected with pRL-CMV (Promega), pBV-Luc BDS-2 3x WT (pBDS-2) (BDS-2 3x WT (p53 binding site) was a gift from Bert Vogelstein (Addgene plasmid #16515; http://n2t.net/addgene:16515; RRID:Addgene_16515; [56])), and either pcDNA3.1(+) as an empty vector control, pcDNA3.Myc-p53 alone or pcDNA3.Myc-p53 in combination with increasing amount pcDNA3.Myc plasmids encoding the indicated p63 variants. The total amount of transfected plasmid DNA was kept constant by addition of pcDNA3.1(+) if necessary. Apart from that, the luciferase reporter displacement assay was performed as the conventional variant described above.

For all luciferase reporter assays presented in this study at least three independent biological replicates were performed. Statistical significance was assessed by ordinary one-way ANOVA followed by Tukey’s post hoc test using Graphpad Prism 8 (Supplementary Table 3).

### DNA-pulldown

100 pmol of biotinylated annealed dsDNA oligonucleotides (Supplementary Table 4) were immobilized on 25 µl Streptavidin Sepharose High Performance affinity resin (Cytiva) in pulldown buffer (50 mM Tris pH 8.0, 150 mM NaCl and 0.1% Tween 20) for 1 h at 4 °C. Simultaneously, Myc-tagged p63 wildtype and mutants were produced by in-vitro translation (#L1170, Promega) from the respective pcDNA3.Myc plasmids. Reaction were incubated for 90 min at 30 °C and afterwards cleared by centrifugation. As an input sample, 5 μl of the reaction were mixed with 95 μl 2x SDS-PAGE sample buffer and boiled. Loaded beads were washed three times with pulldown buffer to remove unbound DNA and incubated with 20 µl in-vitro translated p63 in pulldown buffer with a final volume of 400 µl for 3 h at 4 °C rotating. Bead slurry was transferred into Ultrafree-MC Centrifugal Filter (Merck KGaA) and centrifuged at 250 xg for 1 min. The flow-through was discarded and beads were washed four times with 400 µl ice-cold pulldown buffer by centrifugation. Filters were transferred into a new tubes and bound proteins were eluted by addition of 100 µl boiling 2x SDS-sample buffer followed by centrifugation. Input and pulldown samples were analysed by western blotting and quantified.

For the relative pull-down efficiency each pulldown sample was normalized to the input sample and normalized to p63 wildtype. The DNA pull-down assays were performed as three independent biological replicates. Statistical significance was assessed by ordinary one-way ANOVA followed by Tukey’s post hoc test using Graphpad Prism 8 (Supplementary Table 3).

### RNA Isolation, RT-qPCR and RNA-seq

#### RT-qPCR

Total RNA was extracted using TRIzol reagent (Thermo Fisher Scientific) and cDNA was synthesized using SuperScript Vilo (Thermo Fisher Scientific) and Luna Superscript (New England Biolabs). RT-qPCR was performed using the SYBR Green PCR master mix (Thermo Fisher Scientific) and Luna Sybr (New England Biolabs) in an ABI PRISM 7500 (Thermo Fisher Scientific). Target genes were quantified using specific oligonucleotide primers (Supplementary Table 4) and normalized for human RPLP0 expression. Statistical significance was assessed by ordinary one-way ANOVA followed by Tukey’s post hoc test using Graphpad Prism 8 (Supplementary Table 3).

#### RNA-seq

For the RNA-sequencing, RNA was isolated using the RNAeasy Mini Kit (Qiagen). Total RNA was isolated from three independent experiments and polyA RNA was sequenced at the 3’ end using Quant-Seq FWD 30 mRNA-Seq Kit (Lexogen, Austria). Sequencing was performed using an Illumina Hi-Seq 4000 at the Telethon Institute of Genetics and Medicine (Pozzuoli, Italy). Binary base call files were converted in FASTQ file through bcl2fastq1 (Illumina; version v2.20.0.422). Sequence reads were trimmed using BBDuk software2 (bbmap suite 37.31, https://jgi.doe.gov/data-and-tools/bbtools/bb-tools-user-guide/usage-guide/) to remove adapter sequences, poly-A tails and low-quality end bases (regions with average quality below 6). Alignment was performed with STAR RNA-seq aligner 2.6.0a3 on hg38 reference assembly obtained from Cell Ranger website (Ensembl 93) (https://support.10xgenomics.com/single-cell-gene-expression/software/release-notes/build#mm10_3.0.0). The expression levels of genes were determined with htseq-count 0.9.1 by using cellRanger pre-build genes annotations (Ensembl Assembly 93). All genes having <1 cpm in less than n_min samples and Perc MM reads >20% simultaneously were filtered out. Differential expression analysis was performed using edgeR5. Genes were filtered using the FDR ≤ 0.01 and gene annotation analysis was performed with Metascape Express analysis.

### ChIP-seq and ATAC-seq

ChIP-seq and ATAC-seq experiments were performed as duplicates in Tet-inducible BJ-HDF cells (see above). Each replicate corresponds to an independent round of induction of the same set of polyclonal cell lines.

#### ChIP-seq

4 × 10 ^ 6 cells were cross-linked with 1% formaldehyde in PBS, lysed in 500 μl of SDS lysis Buffer (1% SDS, 10 mM EDTA and 50 mM Tris pH 8.1, 1 mM PMSF, protease inhibitors) for 10 min in ice, and sonicated using a Bandelin Sonopuls device in order to obtain DNA fragments of between 200 and 500 bp in size. Samples were centrifuged 10 min at 4 °C, and supernatant was diluted in ChIP dilution Buffer (0.01% SDS, 1.1% Triton X-100, 1.2 mM EDTA, 16.7 mM Tris-HCl pH 8.1, 167 mM NaCl, 1 mM PMSF, protease inhibitors). Samples were then incubated overnight at 4 °C on a rotating wheel with 4 μg of p63-specific antibodies per sample (Abcam, #124762) and 25 μl of Protein A/G Dynabeads (1:1, #10006D and #10007D, Thermo Fisher Scientific). Beads were washed twice with High Salt Immune Complex Wash Buffer (0.1% SDS, 1% Triton X-100, 2 mM EDTA, 20 mM Tris-HCl pH 8.1, 500 mM NaCl), Low Salt Immune Complex Wash Buffer (0.1% SDS, 1% Triton X-100, 2 mM EDTA, 20 mM Tris-HCl pH 8.1, 150 mM NaCl), LiCl Immune Complex Wash Buffer (0.25 M LiCl, 1% IGEPAL, 1% deoxycholic acid, 1 mM EDTA, 10 mM Tris pH 8.1), and TE Buffer (10 mM Tris-HCl pH 8.0, 1 mM EDTA). Elution from the beads was achieved by incubation in elution buffer (1% SDS, 100 mM NaHCO_3_) for 15 min and cross-link was reversed by adding 0.2 M NaCl and incubating overnight at 65 °C. Samples were incubated for 1 h at 45 °C with proteinase K (#EMR023100, EuroClone) and purified using MinElute PCR purification kit (#28004, Qiagen).

#### ATAC-seq

Fifty-thousand cells were resuspended in lysis buffer (10 mM Tris pH 7.5, 10 mM NaCl, 3 mM MgCl_2_, 0.1% IGEPAL) and centrifuged at 500 x g for 10 min at 4 °C to collect nuclei. Nuclei were placed in 50 μl of Transposase reaction with 2 μl Tagment DNA Enzyme (#20034197, Illumina Tagment DNA Enzyme and Buffer kit, Illumina). Tagmentation was performed incubating samples at 37 °C for 1 h with 650 rpm shaking. To stop tagmentation, clean-up buffer (1 mM NaCl, 0.5 mM EDTA, 1% SDS and 10 µg/µL Proteinase K) was added to samples and reactions were incubated at 40 °C for 30 min with 650 rpm shaking. DNA purification was performed using AMPure XP for PCR Purification (#A63880, Beckman Coulter Life Sciences).

#### Library preparation and sequencing

Purified DNA from ChIP-seq and ATAC-seq experiments was used for library construction using KAPA Hyper Prep Kit (#KK8504, Kapa Biosystems) according to the standard protocol as previously described [35, 57]. The prepared libraries were then sequenced using the NextSeq 500 (Illumina) according to standard Illumina protocols.

#### ChIP-seq and ATAC-seq analyses

ChIP-seq and ATAC-seq fastq files of each condition was mapped to the *Homo sapiens* genome (hg38 build) using Bowtie2 v2.3.4.3. MACS2 peak-calling was used to call peaks from two replicates of each condition, using input as control for ChIP-seq. The average resulting peak files were sorted by -Log10(FDR) and filtered considering only peaks ≥20 and with a fold of enrichment (for ChIP-seq). All heat plot and read tag density figures were generated using the seqMiner program (version 1.3.3) [58]. Peak distribution and clustering were performed using ChIPseeker [59] and bedtools Intersect intervals [60]. De novo motif discovery and motif scanning was performed using MEME-ChIP (version 5.3.1) [61].

p63 motif scanning was performed using MAST (Motif Alignment and Search Tool) [62], a tool of the MEME Suite. Matrices for TP63 binding sites MA0525.1 and MA0525.2 were obtained from the JASPAR database [63] and were used altogether or split into two separate motifs to search for hemi-sites.

### Reporting summary

Further information on research design is available in the Nature Research Reporting Summary linked to this article.

## Supplementary information

Reporting Summary

Supplementary Material

Supplementary Figure S1

Supplementary Figure S2

Supplementary Figure S3

Supplementary Figure S4

Supplementary Figure S5

Supplementary Figure S6

original data files

Supplementary Table S1

Supplementary Table S2

Supplementary Table S3

Supplementary Table S4

## Data Availability

All sequencing data generated during this study have been deposited at GEO (GSE221530).

## References

[1] Yang A, Schweitzer R, Sun D, Kaghad M, Walker N, Bronson RT (1999). p63 is essential for regenerative proliferation in limb, craniofacial and epithelial development. Nature..

[2] Mills AA, Zheng B, Wang XJ, Vogel H, Roop DR, Bradley A (1999). p63 is a p53 homologue required for limb and epidermal morphogenesis. Nature..

[3] Inoue K, Fry EA (2014). Alterations of p63 and p73 in human cancers. Subcell Biochem.

[4] Celli J, Duijf P, Hamel BC, Bamshad M, Kramer B, Smits AP (1999). Heterozygous germline mutations in the p53 homolog p63 are the cause of EEC syndrome. Cell..

[5] Rinne T, Brunner HG, van Bokhoven H (2007). p63-associated disorders. Cell Cycle.

[6] Osterburg C, Osterburg S, Zhou H, Missero C, Dotsch V (2021). Isoform-specific roles of mutant p63 in human diseases. Cancers (Basel).

[7] Bertola DR, Kim CA, Albano LMJ, Scheffer H, Meijer R, van Bokhoven H (2004). Molecular evidence that AEC syndrome and Rapp-Hodgkin syndrome are variable expression of a single genetic disorder. Clinical Genetics.

[8] Prontera P, Escande F, Cocchi G, Donti E, Martini A, Sensi A (2008). An intermediate phenotype between hay-wells and rapp-hodgkin syndromes in a patient with a novel P63 mutation: confirmation of a variable phenotypic spectrum with a common aetiology. Genet Counsel.

[9] McGrath JA, Duijf PH, Doetsch V, Irvine AD, de Waal R, Vanmolkot KR (2001). Hay-Wells syndrome is caused by heterozygous missense mutations in the SAM domain of p63. Hum Mol Genet.

[10] Chi SW, Ayed A, Arrowsmith CH (1999). Solution structure of a conserved C-terminal domain of p73 with structural homology to the SAM domain. EMBO J.

[11] Serber Z, Lai HC, Yang A, Ou HD, Sigal MS, Kelly AE (2002). A C-terminal inhibitory domain controls the activity of p63 by an intramolecular mechanism. Mol Cell Biol.

[12] Russo C, Osterburg C, Sirico A, Antonini D, Ambrosio R, Wurz JM (2018). Protein aggregation of the p63 transcription factor underlies severe skin fragility in AEC syndrome. Proc Natl Acad Sci U S A.

[13] Sutton VR, van Bokhoven H TP63-Related Disorders. In: Adam MP, Mirzaa GM, Pagon RA, Wallace SE, Bean LJH, Gripp KW, et al., editors. GeneReviews((R)). Seattle (WA) 1993. 20556892

[14] Bullock AN, Henckel J, DeDecker BS, Johnson CM, Nikolova PV, Proctor MR (1997). Thermodynamic stability of wild-type and mutant p53 core domain. Proc Natl Acad Sci U S A.

[15] Joerger AC, Ang HC, Fersht AR (2006). Structural basis for understanding oncogenic p53 mutations and designing rescue drugs. Proc Natl Acad Sci U S A.

[16] Joerger AC, Fersht AR (2007). Structure-function-rescue: the diverse nature of common p53 cancer mutants. Oncogene..

[17] Bullock AN, Henckel J, Fersht AR (2000). Quantitative analysis of residual folding and DNA binding in mutant p53 core domain: definition of mutant states for rescue in cancer therapy. Oncogene..

[18] Logotheti S, Pavlopoulou A, Marquardt S, Takan I, Georgakilas AG, Stiewe T (2022). p73 isoforms meet evolution of metastasis. Cancer Metast Rev.

[19] Nemajerova A, Amelio I, Gebel J, Dotsch V, Melino G, Moll UM (2018). Non-oncogenic roles of TAp73: from multiciliogenesis to metabolism. Cell Death Differ.

[20] Osterburg C, Dotsch V (2022). Structural diversity of p63 and p73 isoforms. Cell Death Differ.

[21] Dehner A, Klein C, Hansen S, Muller L, Buchner J, Schwaiger M (2005). Cooperative binding of p53 to DNA: regulation by protein-protein interactions through a double salt bridge. Angew Chem Int Ed Engl.

[22] Klein C, Georges G, Kunkele KP, Huber R, Engh RA, Hansen S (2001). High thermostability and lack of cooperative DNA binding distinguish the p63 core domain from the homologous tumor suppressor p53. J Biol Chem.

[23] Coutandin D, Lohr F, Niesen FH, Ikeya T, Weber TA, Schafer B (2009). Conformational stability and activity of p73 require a second helix in the tetramerization domain. Cell Death Differ.

[24] Joerger AC, Rajagopalan S, Natan E, Veprintsev DB, Robinson CV, Fersht AR (2009). Structural evolution of p53, p63, and p73: implication for heterotetramer formation. Proc Natl Acad Sci U. S. A.

[25] Gebel J, Luh LM, Coutandin D, Osterburg C, Lohr F, Schafer B (2016). Mechanism of TAp73 inhibition by DeltaNp63 and structural basis of p63/p73 hetero-tetramerization. Cell Death Differ.

[26] Rinne T, Hamel B, van Bokhoven H, Brunner HG (2006). Pattern of p63 mutations and their phenotypes-update. Am J Med Genet.

[27] Kehrloesser S, Osterburg C, Tuppi M, Schafer B, Vousden KH, Dotsch V (2016). Intrinsic aggregation propensity of the p63 and p73 TI domains correlates with p53R175H interaction and suggests further significance of aggregation events in the p53 family. Cell Death Differ.

[28] Nikolova PV, Henckel J, Lane DP, Fersht AR (1998). Semirational design of active tumor suppressor p53 DNA binding domain with enhanced stability. P Natl Acad Sci U. S. A.

[29] Khoo KH, Andreeva A, Fersht AR (2009). Adaptive evolution of p53 thermodynamic stability. J Mol Biol.

[30] Chen Y, Mistry DS, Sen GL (2014). Highly rapid and efficient conversion of human fibroblasts to keratinocyte-like cells. J Invest Dermatol.

[31] Ferone G, Mollo MR, Thomason HA, Antonini D, Zhou HQ, Ambrosio R (2013). p63 control of desmosome gene expression and adhesion is compromised in AEC syndrome. Hum Mol Genet.

[32] Romano RA, Birkaya B, Sinha S (2007). A functional enhancer of keratin14 is a direct transcriptional target of deltaNp63. J Invest Dermatol.

[33] Lin-Shiao E, Lan Y, Welzenbach J, Alexander KA, Zhang Z, Knapp M (2019). p63 establishes epithelial enhancers at critical craniofacial development genes. Sci Adv.

[34] McDade SS, Henry AE, Pivato GP, Kozarewa I, Mitsopoulos C, Fenwick K (2012). Genome-wide analysis of p63 binding sites identifies AP-2 factors as co-regulators of epidermal differentiation. Nucl Acids Res.

[35] Qu J, Yi G, Zhou H (2019). p63 cooperates with CTCF to modulate chromatin architecture in skin keratinocytes. Epigenetics Chromatin.

[36] Sethi I, Sinha S, Buck MJ (2014). Role of chromatin and transcriptional co-regulators in mediating p63-genome interactions in keratinocytes. BMC Genomics.

[37] Chen C, Gorlatova N, Kelman Z, Herzberg O (2011). Structures of p63 DNA binding domain in complexes with half-site and with spacer-containing full response elements. P Natl Acad Sci U. S. A.

[38] Chen C, Gorlatova N, Herzberg O (2012). Pliable DNA conformation of response elements bound to transcription factor p63. J Biol Chem.

[39] Straub WE, Weber TA, Schafer B, Candi E, Durst F, Ou HD (2010). The C-terminus of p63 contains multiple regulatory elements with different functions. Cell Death Dis.

[40] Huang YP, Wu G, Guo Z, Osada M, Fomenkov T, Park HL (2004). Altered sumoylation of p63alpha contributes to the split-hand/foot malformation phenotype. Cell Cycle.

[41] Rossi M, De Simone M, Pollice A, Santoro R, La Mantia G, Guerrini L (2006). Itch/AIP4 associates with and promotes p63 protein degradation. Cell Cycle.

[42] Ethayathulla AS, Tse PW, Monti P, Nguyen S, Inga A, Fronza G (2012). Structure of p73 DNA-binding domain tetramer modulates p73 transactivation. P Natl Acad Sci U. S. A.

[43] Pagano B, Jama A, Martinez P, Akanho E, Bui TT, Drake AF (2013). Structure and stability insights into tumour suppressor p53 evolutionary related proteins. PLoS One.

[44] Patel S, Bui TT, Drake AF, Fraternali F, Nikolova PV (2008). The p73 DNA binding domain displays enhanced stability relative to its homologue, the tumor suppressor p53, and exhibits cooperative DNA binding. Biochemistry..

[45] Sievers F, Wilm A, Dineen D, Gibson TJ, Karplus K, Li W (2011). Fast, scalable generation of high-quality protein multiple sequence alignments using Clustal Omega. Mol Syst Biol.

[46] Goujon M, McWilliam H, Li WZ, Valentin F, Squizzato S, Paern J (2010). A new bioinformatics analysis tools framework at EMBL-EBI. Nucl Acids Res.

[47] Coutandin D, Osterburg C, Srivastav RK, Sumyk M, Kehrloesser S, Gebel J (2016). Quality control in oocytes by p63 is based on a spring-loaded activation mechanism on the molecular and cellular level. Elife..

[48] Morgenstern JP, Land H (1990). Advanced mammalian gene-transfer - high titer retroviral vectors with multiple-drug selection markers and a complementary helper-free packaging cell-line. Nucl Acids Res.

[49] Maherali N, Ahfeldt T, Rigamonti A, Utikal J, Cowan C, Hochedlinger K (2008). A high-efficiency system for the generation and study of human induced pluripotent stem cells. Cell Stem Cell.

[50] Butler JS, Loh SN (2003). Structure, function, and aggregation of the zinc-free form of the p53 DNA binding domain. Biochemistry..

[51] Solyom Z, Schwarten M, Geist L, Konrat R, Willbold D, Brutscher B (2013). BEST-TROSY experiments for time-efficient sequential resonance assignment of large disordered proteins. J Biomol NMR.

[52] Enthart A, Klein C, Dehner A, Coles M, Gemmecker G, Kessler H (2016). Solution structure and binding specificity of the p63 DNA binding domain. Sci Rep.

[53] Timofeev O, Koch L, Niederau C, Tscherne A, Schneikert J, Klimovich M (2020). Phosphorylation control of p53 DNA-binding cooperativity balances tumorigenesis and aging. Cancer Res.

[54] Takahashi K, Yamanaka S (2006). Induction of pluripotent stem cells from mouse embryonic and adult fibroblast cultures by defined factors. Cell..

[55] Pitzius S, Osterburg C, Gebel J, Tascher G, Schafer B, Zhou H (2019). TA*p63 and GTAp63 achieve tighter transcriptional regulation in quality control by converting an inhibitory element into an additional transactivation domain. Cell Death Dis.

[56] Hermeking H, Lengauer C, Polyak K, He TC, Zhang L, Thiagalingam S (1997). 14-3-3 sigma is a p53-regulated inhibitor of G2/M progression. Mol Cell.

[57] Qu J, Tanis SEJ, Smits JPH, Kouwenhoven EN, Oti M, van den Bogaard EH (2018). Mutant p63 affects epidermal cell identity through rewiring the enhancer landscape. Cell Rep.

[58] Ye T, Krebs AR, Choukrallah MA, Keime C, Plewniak F, Davidson I (2011). seqMINER: an integrated ChIP-seq data interpretation platform. Nucl Acids Res.

[59] Yu GC, Wang LG, He QY (2015). ChIPseeker: an R/Bioconductor package for ChIP peak annotation, comparison and visualization. Bioinformatics..

[60] Quinlan AR, Hall IM (2010). BEDTools: a flexible suite of utilities for comparing genomic features. Bioinformatics..

[61] Machanick P, Bailey TL (2011). MEME-ChIP: motif analysis of large DNA datasets. Bioinformatics..

[62] Bailey TL, Gribskov M (1998). Combining evidence using *p*-values: application to sequence homology searches. Bioinformatics..

[63] Castro-Mondragon JA, Riudavets-Puig R, Rauluseviciute I, Lemma RB, Turchi L, Blanc-Mathieu R (2022). JASPAR 2022: the 9th release of the open-access database of transcription factor binding profiles. Nucl Acids Res.

