# Supplementary material for: Disease-related p63 DBD mutations impair DNA binding by distinct mechanisms and varying degree
Source: Cell Death Dis. 2023 Apr 18;14(4):274. doi: 10.1038/s41419-023-05796-y (PMC10113246; doi:10.1038/s41419-023-05796-y)
Supplement: Supplementary file 2 — Supplementary Material [file 41419_2023_5796_MOESM2_ESM.docx]

**Supplementary Material**

**Supplementary figure legends**

**Supplementary Figure 1.**

**A** Sequence alignment of the p63 DBD (aa 90-294) and the p53 DBD (aa 119-325). The DBDs comprise ten β-strands (S1 to S10), three α-helices (Ha, H1 and H2) as well as four loops involved in DNA binding (L1, L2A, L2B and L3). These elements are depicted above the alignment. DNA-contacting and zinc-coordinating residues are marked by brown and purple frames, respectively. Amino acids responsible for differences in the thermodynamic stability are indicated by grey boxes. In the p63 DBD sequence mutations related to ELA syndrome mutations are coloured in blue, SHFM4 mutations in green and AEC/RHS mutations in red. Hotspot mutations are highlighted in bold. **B** Superimposition of the p63 and p53 DBD bound to DNA (PDB: 3QYN and 2AC0). Structural elements are colour-coded according to (**A**). Relevant helices and loops are highlighted with labels. DNA is shown in light green and the zinc ion as a purple sphere. **C** The hydrophobic core (residues depicted as spheres) of the p63 DBD is optimally packed in contrast to p53, which is the basis for its metastability [1]. The p53 core has small cavities due to suboptimal packaging and three unfavourable residues with partially polar side chains (M133, Y236 and T253 (blue spheres) and V203 (purple stick)). The corresponding ones in p63 are residues of optimal size and hydrophobicity (L162, F267 and I284 (blue spheres) and A234 (purple stick)). An additional difference is the surface exposed N268 in p53, which is a charged amino acid R299 in p63 (yellow stick). Efforts in stabilizing the p53 DBD lead to several thermostabilizing (TS) mutations (shown in red), comparable with the p63 core (M133L, V203A, Y236F, T253I and N268D). Exception is the N239Y exchange in the DNA binding interface (light red stick). DNA is shown in orange. **D** The data set of p63 disease mutations visualized in Fig. 1B was analysed for the frequency of individual substitutions of all mutational hotspots (Supplementary Table S1). n equals the number of families and + marks substitutions related to CpG sites. **E** Data of somatic p53 mutations in human cancers were extracted from the COSMIC database (version 90; https://cancer.sanger.ac.uk/cosmic; [2]) and analysed for frequency of mis- and nonsense mutation occurring for each residue in p53 (n=28,608 cases) (Supplementary Table S1). Mutation frequencies are plotted as bars along the p53 protein sequence aligned to the schematic domain architecture. The positions of the nine mutational hotspots (missense: R175, H179, Y220, G245, R248, R249, R273 and R282 (black); nonsense: R213 (grey)) are highlighted with labels. TAD: Transactivation domain; DBD: DNA binding domain; OD: oligomerization domain. **F** Mutation frequency was calculated for the p53 DBD individually (n=23,767 cases) and blotted with the indicated colour code on the DBD structure (PDB: 2AC0) (Supplementary Table S1). The eight mutational hotspots are depicted as sticks and highlighted with labels. DNA is shown in light green and the zinc ion as a purple sphere. **G** The data set of human p53 cancer mutations visualized in (**E**) was analysed for the frequency of individual substitutions of all mutational hotspots (Supplementary Table S1). n equals the number of unique cases and + marks substitutions related to CpG sites.

**Supplementary Figure 2.**

**A** Luciferase reporter assay of ΔNp63α and p53 WT and the indicated ELA and cancer DBD mutants using the KRT14 and p21 promoter. H1299 cells were transiently transfected with the respective luciferase reporter plasmids and the Myc-tagged proteins. The bar diagram shows the mean activity relative to WT and error bars the corresponding SD (n=3). **B** Expression levels of the transiently transfected proteins in the luciferase reporter assay from (**A**) were determined by WB using an α-Myc antibody (Supplementary Fig. S7). GAPDH served as a loading control. **C** Luciferase reporter assay of ΔNp63α WT and the indicated ELA DBD mutants using the KRT14 promoter. H1299 cells were transiently transfected with the respective luciferase reporter plasmids and the Myc-tagged proteins. The bar diagram shows the mean activity relative to WT and error bars the corresponding SD (n=3). **D** Expression levels of the transiently transfected proteins in the luciferase reporter assay from (**C**) were determined by WB using an α-Myc antibody (Supplementary Fig. S7). GAPDH served as a loading control. **E** Luciferase reporter assay of ΔNp63α WT and the indicated ADULT and SHFM4 DBD mutants using the KRT14 promoter. H1299 cells were transiently transfected with the respective luciferase reporter plasmids and the Myc-tagged proteins. The bar diagram shows the mean activity relative to WT and error bars the corresponding SD (n=3). **F** Expression levels of the transiently transfected proteins in the luciferase reporter assay from (**E**) were determined by WB using an α-Myc antibody (Supplementary Fig. S7). GAPDH served as a loading control. **G** Luciferase reporter assay of ΔNp63α WT and the indicated ELA DBD mutants using the KRT14 promoter. H1299 cells were transiently transfected with the respective luciferase reporter plasmids and the Myc-tagged proteins. The bar diagram shows the mean activity relative to WT and error bars the corresponding SD (n=3). **H** Expression levels of the transiently transfected proteins in the luciferase reporter assay from (**G**) were determined by WB using an α-Myc antibody (Supplementary Fig. S7). GAPDH served as a loading control. **I** DNA pull-down assay of ΔNp63α WT and indicated ELA and artificial mutants with the REs of the human KRT14 and p21 promoter as bait. Myc-tagged proteins were *in-vitro* translated using RRL. Input (IP) and pull-down (PD) samples were analysed by WB using an α-Myc antibody. For the relative pull-down efficiency each PD signal was normalized to the IP signal and p63 WT was set to 1. The bar diagram shows the mean values and error bars the corresponding SD (n=3). **J** Representative WB of the DNA pull-down assay from (**I**) (Supplementary Fig. S7). **K** Representative SPR sensograms of purified p63 DBD-TD WT and indicated mutants binding to a 20bp RE of the p21 promoter immobilized on a streptavidin (SA) chip. **L** SPR affinity curves of purified p63 DBD WT in its holo and apo form as well as the indicated mutants binding to a 20bp consensus sequence (CS) immobilized on a streptavidin (SA) chip. All measurements were conducted on the same chip. Data points were extracted by equilibrium analysis of sensograms (**N**) and plotted. Binding curves were fitted with a non-linear, least squares regression using a single-exponential one-site binding model with Hill slope (Supplementary Table S3). **M** DNA binding affinities of purified p63 DBD WT and indicated mutants derived from the fitted binding curves (**L**). The bar diagrams shows the equilibrium dissociation constants (K_D_) with the error bars corresponding to the 95% confidence interval. **N** SPR sensograms from (**L**). **A**, **C**, **E**, **G**, **I** Statistical significance was assessed by ordinary one-way ANOVA followed by Tukey’s post hoc test (Supplementary Table S3). n.s. *P* > 0.05, * *P* ≤ 0.05, ** *P* ≤ 0.01, *** *P* ≤ 0.001, **** *P* ≤ 0.0001. **A**-**K**, **N** Mutations related to ELA syndrome are coloured in blue, mutations related to SHFM4 in green and artificial mutants in brown. Hotspot mutations are highlighted in bold.

**Supplementary Figure 3.**

**A** Zinc content assay of the purified p63 WT DBD in its holo and apo form. The p63 WT DBD is initially purified in the zinc-loaded holo form. Subsequently, zinc is removed by incubation at low pH in presence of EDTA to obtain the zinc-free apo form. For the assay, bound zinc is released from the DBD zinc finger by an alkylation agent and the concentration is determined by a colorimetric analysis with the zinc binding dye PAR. The zinc concentration is referenced to the protein concentration. The bar diagram shows the mean fraction of DBDs in the holo form and error bars the corresponding SD (n=3). Statistical significance was assessed by an unpaired t-test (Supplementary Table S3). n.s. *P* > 0.05, * *P* ≤ 0.05, ** *P* ≤ 0.01, *** *P* ≤ 0.001, **** *P* ≤ 0.0001. **B** Overlay of ^1^H-^15^N BEST TROSY HSQC spectra of uniformly labelled p63 DBD in holo (black) and apo form (red) recorded at 600 MHz and a sample temperature of 298 K. **C** Analysis of peaks from the NMR spectra in (**B**). The ^1^H-^15^N BEST-TROSY HSQC spectrum of the holo form was assigned using spectra recorded of a ^15^N and ^13^C uniformly labelled holo DBD. The peaks of the apo form were assigned based on the holo DBD spectrum. Peaks were subdivided in three groups based on the comparison between the holo and apo form, either showing no or only minor shifts (green), being lost in the apo form (red) or not unambiguously assignable (grey). The analysis was visualized by colouring the corresponding residues in a structure of the p63 DBD in its holo form (PDB: 3QYN). DNA is shown in orange and the zinc ion as a purple sphere.

**Supplementary Figure 4.**

**A** Melting temperatures of the purified p63 WT and mutant DBDs in their holo and apo form measured by TSA. The p53 WT and R175H DBD serve as a reference. The apo form of I186F DBD was excluded due to precipitation. The bar diagram shows the mean melting temperature and error bars the corresponding SD (n=3). The red line marks the melting temperature of the p53 R175H DBD and the orange line 37°C. **B** BN-PAGE of p53 WT and indicated cancer mutants. H1299 cells were transiently transfected with the Myc-tagged proteins. Cell lysates were subsequently analysed by BN-PAGE (upper panel) and SDS-PAGE (lower panel) followed by WB using α-Myc antibody (Supplementary Fig. S7). Oligomeric states are indicated by ‘d’ (dimer) and ‘t’ (tetramer), ‘a’ marks high molecular weight species corresponding to aggregates. **C** BN-PAGE of ΔNp63α WT and indicated ELA and artificial DBD mutants. All analysed proteins carry the V603D mutant which abolishes background aggregation caused by the intrinsic low aggregation propensity of the native TID [3]. Cell lysates were subsequently analysed by BN-PAGE (upper panel) and SDS-PAGE (lower panel) followed by WB using α-Myc antibody (Supplementary Fig. S7). Oligomeric states are indicated by ‘m’ (monomer), ‘d’ (dimer) and ‘t’ (tetramer), while ‘a’ marks high molecular weight species corresponding to aggregates. **D** BN-PAGE of p53 WT and indicated cancer mutants with a native or thermostabilised (TS) DBD. H1299 cells were transiently transfected with the Myc-tagged proteins. Cell lysates were subsequently analysed by BN-PAGE (upper panel) and SDS-PAGE (lower panel) followed by WB using α-Myc antibody (Supplementary Fig. S7). Oligomeric states are indicated by ‘d’ (dimer) and ‘t’ (tetramer), ‘a’ marks high molecular weight species corresponding to aggregates. **E** Luciferase reporter assay of p53 WT and the indicated cancer mutants carrying thermostabilizing mutants using the p21 promoter. H1299 cells were transiently transfected with the respective luciferase reporter plasmids and the Myc-tagged proteins. The bar diagram shows the mean activity relative to WT and error bars the corresponding SD (n=3). **F** Expression levels of the transiently transfected proteins in the luciferase reporter assay from (**E**) were determined by WB using an α-Myc antibody (Supplementary Fig. S7). GAPDH served as a loading control.

**D**-**F** 3TS: M133L V203A N268D; 5TS: M133L V203A Y236F T253I N268D; 6TS: M133L V203A Y236F N239Y T253I N268D. **A, E** Statistical significance was assessed by ordinary one-way ANOVA followed by Tukey’s post hoc test (Supplementary Table S3). n.s. *P* > 0.05, * *P* ≤ 0.05, ** *P* ≤ 0.01, *** *P* ≤ 0.001, **** *P* ≤ 0.0001. **A**-**F** Mutations related to ELA syndrome are coloured in blue, mutations related to SHFM4 in green and artificial mutants in brown. Hotspot mutations are highlighted in bold.

**Supplementary Figure 5.**

**A** KRT14 immunofluorescence staining of HDFs transduced with KLF4 alone (CTR) or together with WTp63 or the indicated p63 mutants (green). Nuclei are stained with DAPI (blue). Scale bar (white), 50 μm. Mutations related to ELA syndrome are coloured in blue and mutations related to SHFM4 in green. Hotspot mutations are highlighted in bold. **B** Volcano plots showing differential gene expression of the indicated p63 mutants versus control lacking p63. Differentially regulated genes are represented.

**Supplementary Figure 6.**

**A** Expression of p63, KRT14 and beta-actin in HDF expressing inducible KLF4 and the indicated p63 (wild type or mutant) after doxycycline (doxy) administration for 72 h was assessed by WB (Supplementary Fig. S7). **B** Venn diagram showing overlap of ATAC-seq (as in Fig. 6A) and ChIP-seq peaks (green) in HDF expressing p63 WT or the indicated DBD mutants. **C** Venn diagram showing the overlap among genomic regions bound by WTp63 or mutant p63 identified by ChIP-seq. **D** Top two *de novo* discovery motifs using STREME performed on 100bp spanning the ChIP-seq peaks (-Log10FDR ≥ 20) of p63 WT and the indicated DBD mutants. E-value, frequency of the motif (sites), known motifs, and the distribution are shown. **E** p63 binding sites or hemi-sites were identified in the top scoring 100 sequences (100bp around the summit (-Log10FDR ≥ 20)) using MAST. **F** ATAC-seq and ChIP-seq profiles of p63 WT and EEC mutants in DSP genomic region. Visualization of (bottom to top): ChIP-seq profiles of H3K27ac in normal human epidermal keratinocytes (NHEK); candidate cis-Regulatory Elements (cCREs) in the human genome; GENCODE v32 gene annotation; ChIP-seq in HDF expressing inducible p63 WT or indicated DBD mutants; ATAC-seq in HDF expressing inducible p63 WT or KLF4 only (empty).

**Additional supplementary material**

**Supplementary Figure 7.**

Uncropped WB images.

**Supplementary Table 1.**

Detailed analyses of p63 and p53 mutations extracted from published patient reports and the COSMIC database, respectively. In consensus with the literature the standard amino acid numbering in this manuscript is based on the TAp63 isoform. But for clarity purposes, this table also includes numbering in reference to the TA*p63 and ΔNp63 isoforms, because ΔNp63α is the relevant isoform in context of mutant p63-related developmental diseases and mutations specific for the TA*p63 and ΔNp63 isoforms were reported. Additionally, TA*p63 and ΔNp63 numbering has been irregularly used in the literature as well.

**Supplementary Table 2.**

Comparison of p63 and p53 mutations.

**Supplementary Table 3.**

Summary of statistical analyses and fits performed using the Graphpad software.

**Supplementary Table 4.**

List of oligonucleotides used for RT-qPCR, DNA pulldown and SPR experiments.

**References**

1. Enthart, A., et al., *Solution structure and binding specificity of the p63 DNA binding domain.* Sci Rep, 2016. **6**: p. 26707.

2. Tate, J.G., et al., *COSMIC: the Catalogue Of Somatic Mutations In Cancer.* Nucleic Acids Research, 2019. **47**(D1): p. D941-D947.

3. Kehrloesser, S., et al., *Intrinsic aggregation propensity of the p63 and p73 TI domains correlates with p53R175H interaction and suggests further significance of aggregation events in the p53 family.* Cell Death Differ, 2016. **23**(12): p. 1952-1960.
