# Supplementary figures and images for: Disease-related p63 DBD mutations impair DNA binding by distinct mechanisms and varying degree
Source: Cell Death Dis. 2023 Apr 18;14(4):274. doi: 10.1038/s41419-023-05796-y (PMC10113246; doi:10.1038/s41419-023-05796-y)

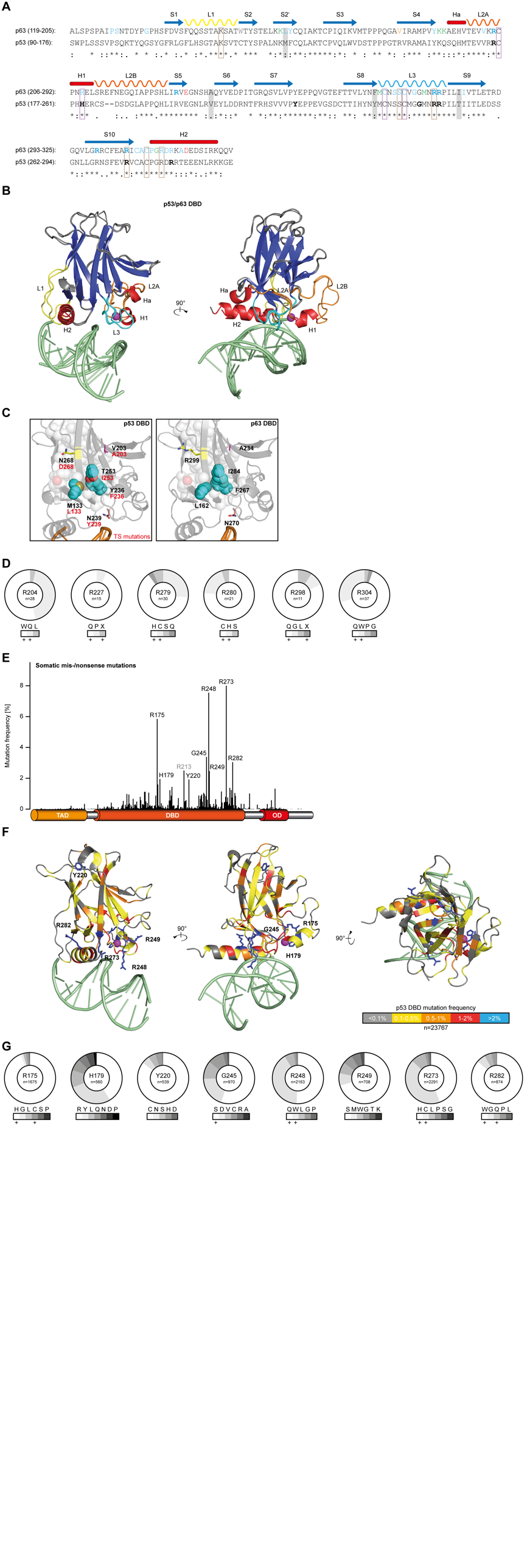

Supplement: Supplementary file 3 — Supplementary Figure S1 [file 41419_2023_5796_MOESM3_ESM.png]

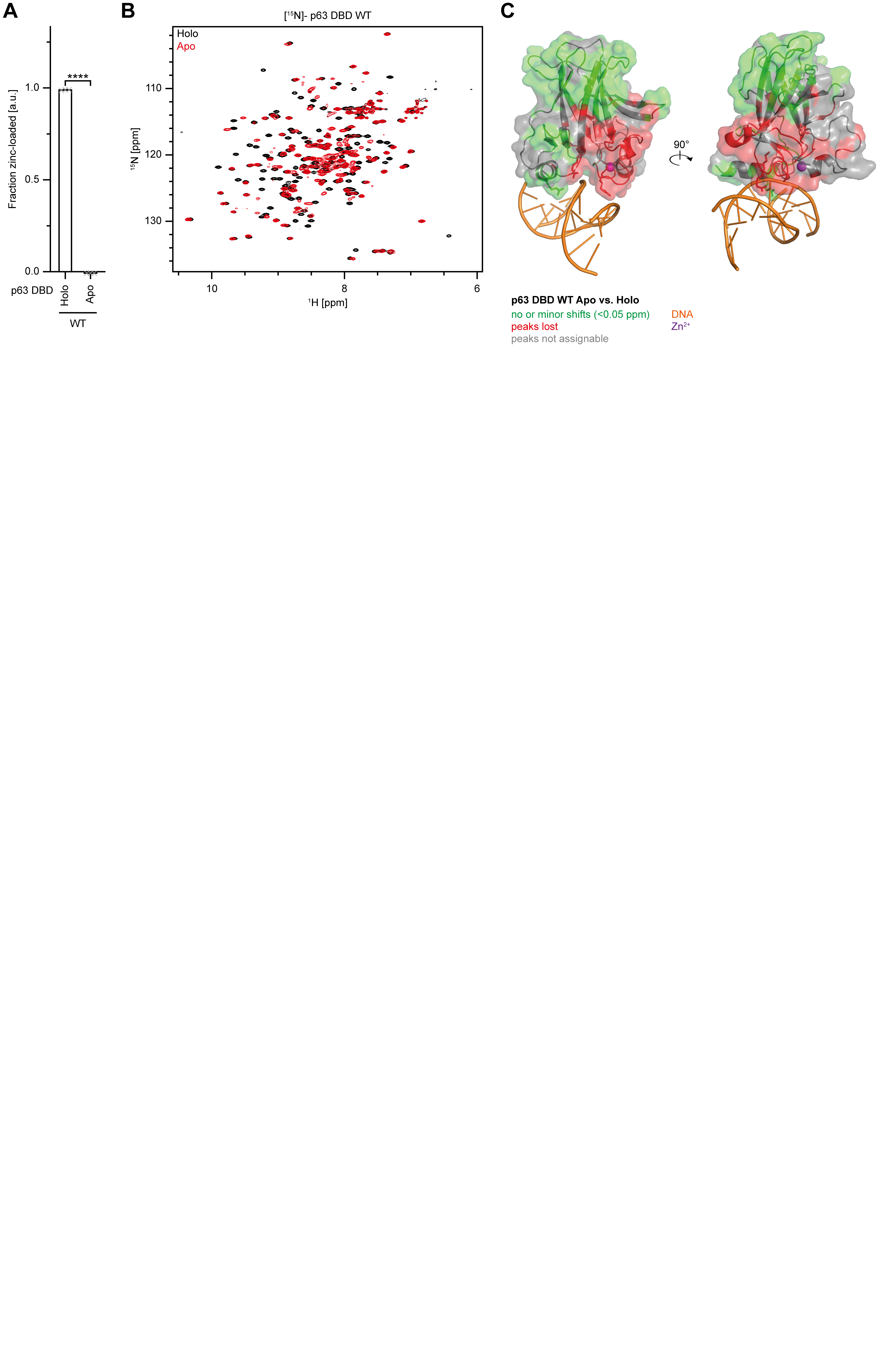

Supplement: Supplementary file 5 — Supplementary Figure S3 [file 41419_2023_5796_MOESM5_ESM.png]

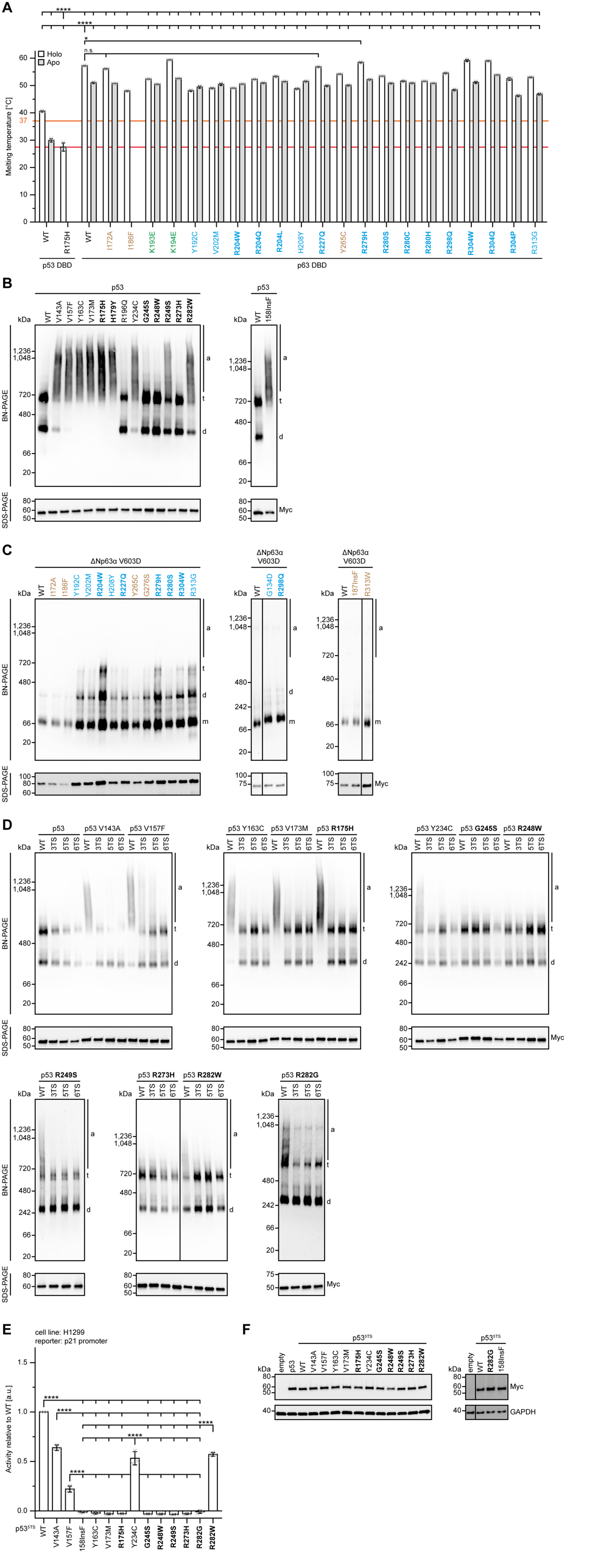

Supplement: Supplementary file 6 — Supplementary Figure S4 [file 41419_2023_5796_MOESM6_ESM.png]

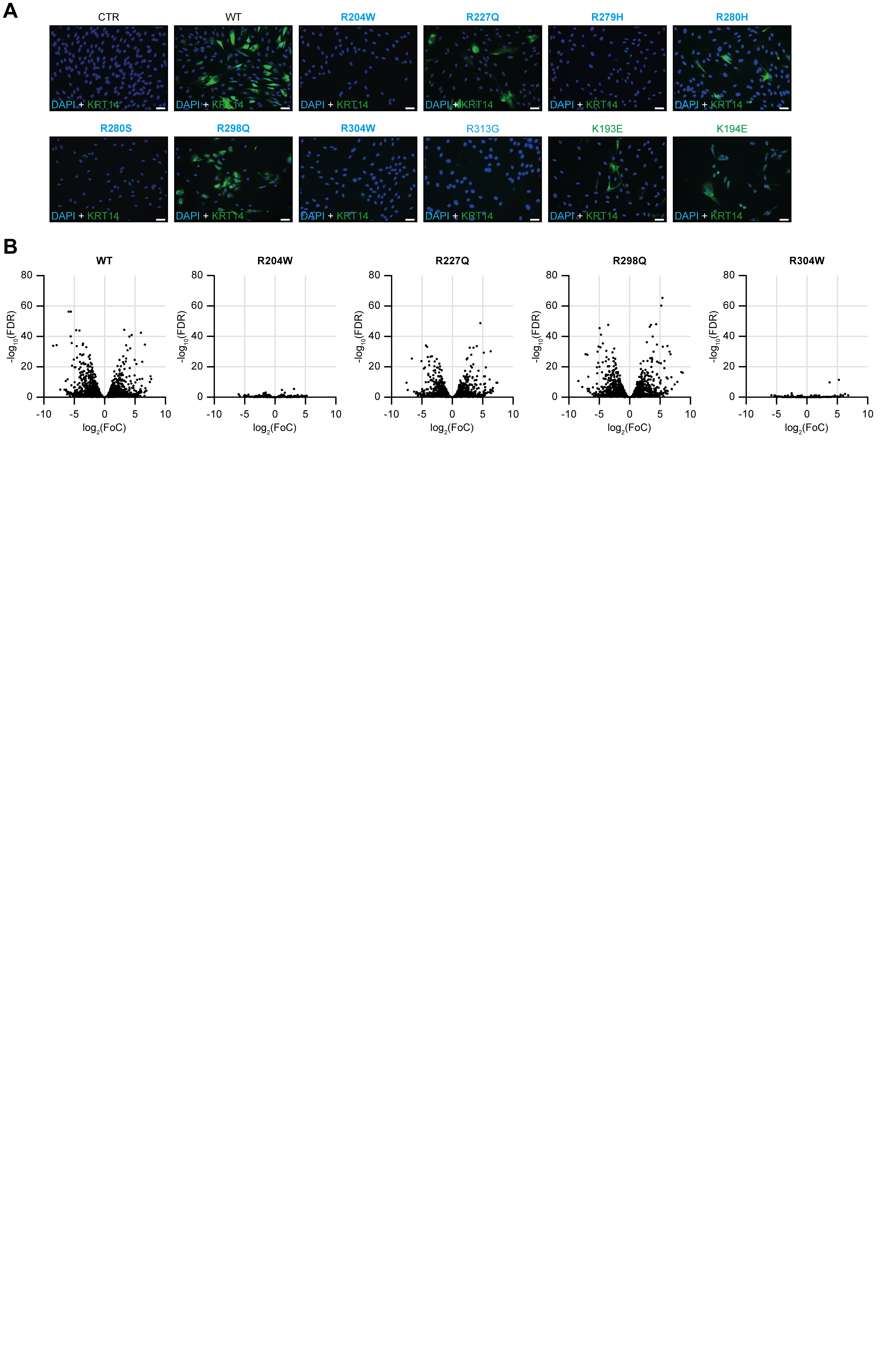

Supplement: Supplementary file 7 — Supplementary Figure S5 [file 41419_2023_5796_MOESM7_ESM.png]

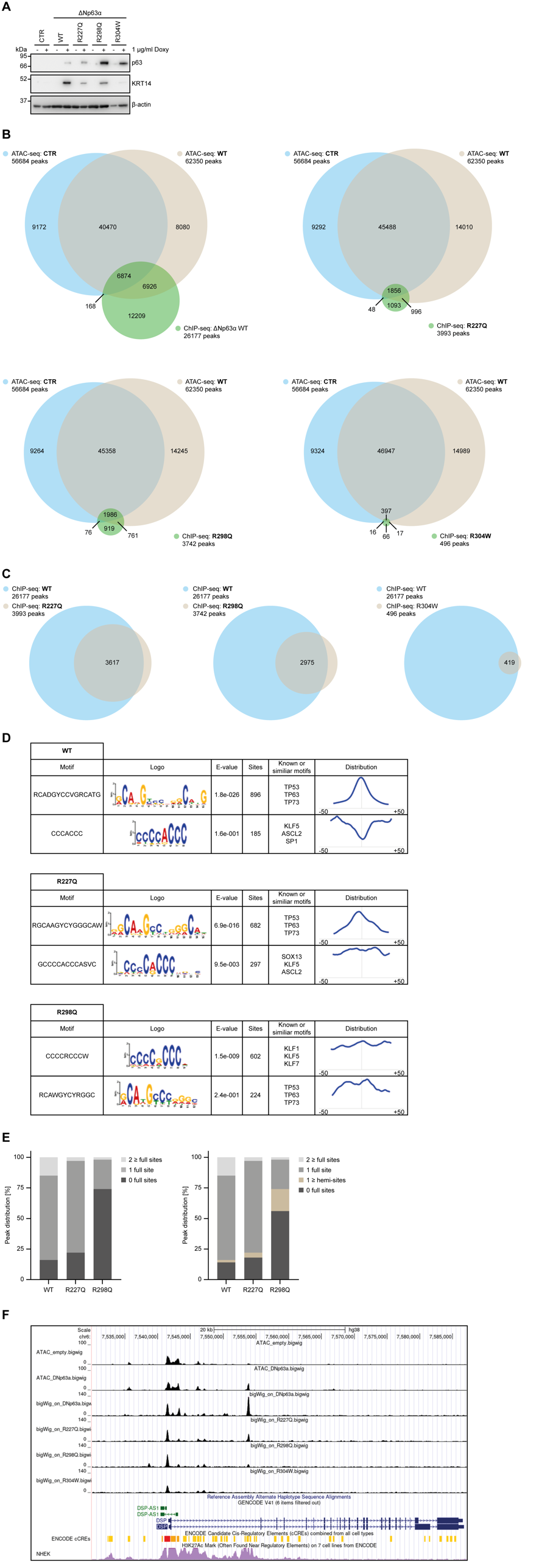

Supplement: Supplementary file 8 — Supplementary Figure S6 [file 41419_2023_5796_MOESM8_ESM.png]
