# Supplementary material for: Disease-related p63 DBD mutations impair DNA binding by distinct mechanisms and varying degree
Source: Cell Death Dis. 2023 Apr 18;14(4):274. doi: 10.1038/s41419-023-05796-y (PMC10113246; doi:10.1038/s41419-023-05796-y)
Supplement: Supplementary file 9 — original data files [file 41419_2023_5796_MOESM9_ESM.pdf]

Figure 2C

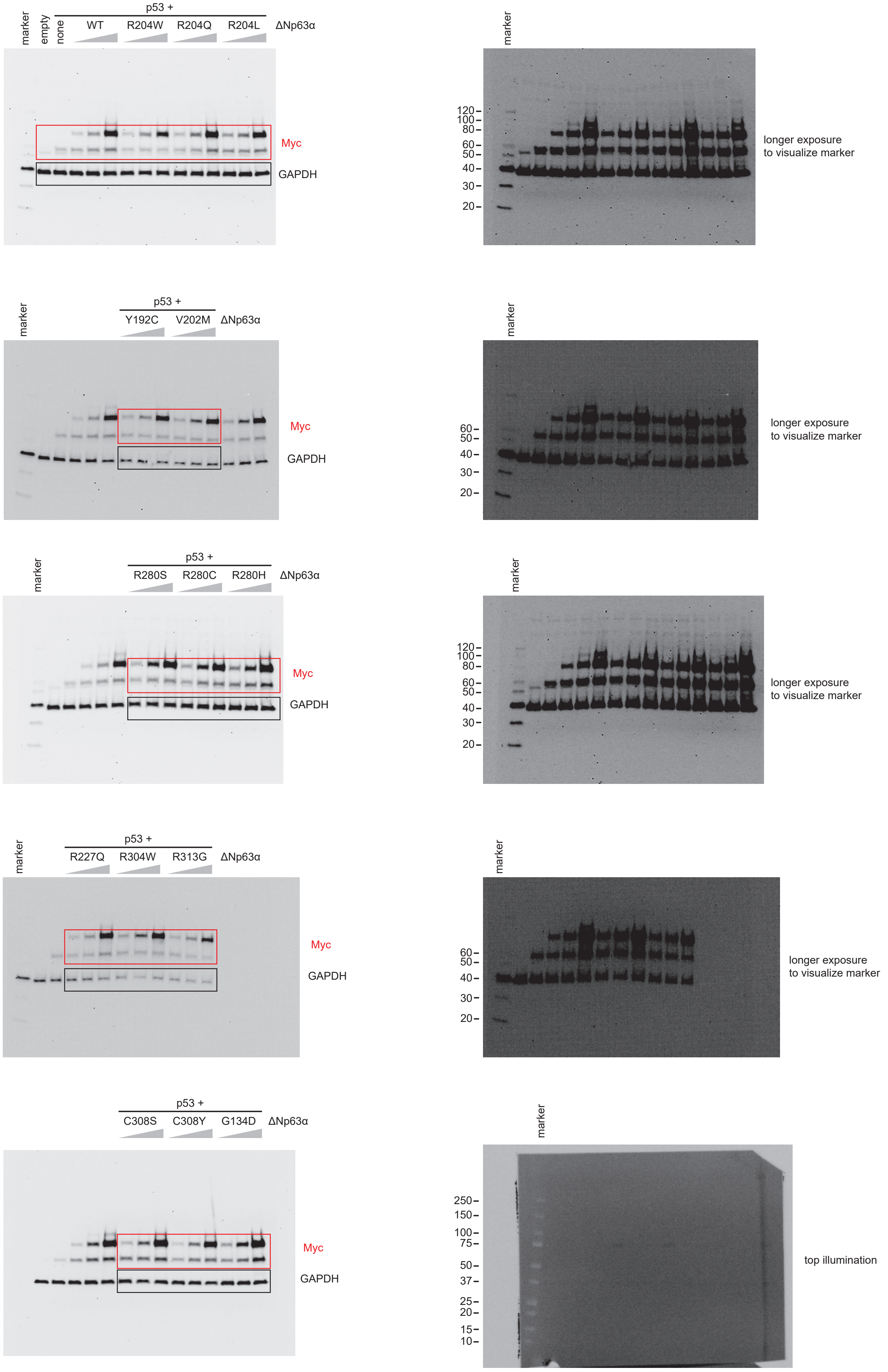

marker
empty
none
p53 +
WT
R204W
R204Q
R204L
ΔNp63α
Myc
GAPDH
120
100
80
60
50
40
30
20
longer exposure
to visualize marker
Y192C
V202M
R280S
R280C
R280H
R227Q
R304W
R313G
C308S
C308Y
G134D
250
150
75
37
25
15
10
top illumination

Figure 2C - continued

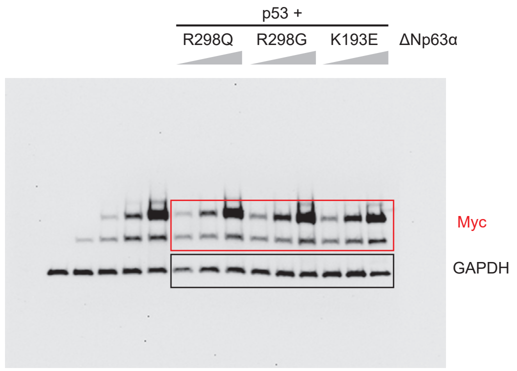

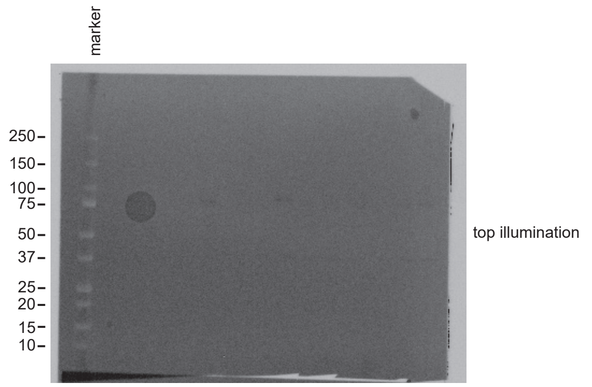

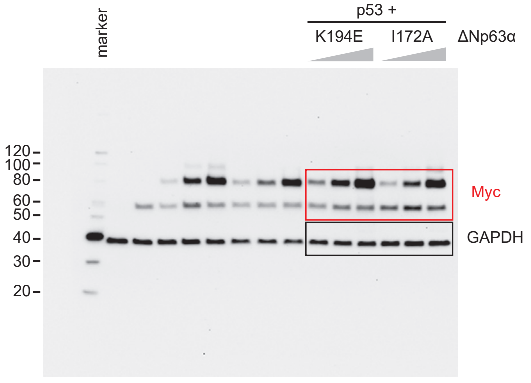

Figure S2B

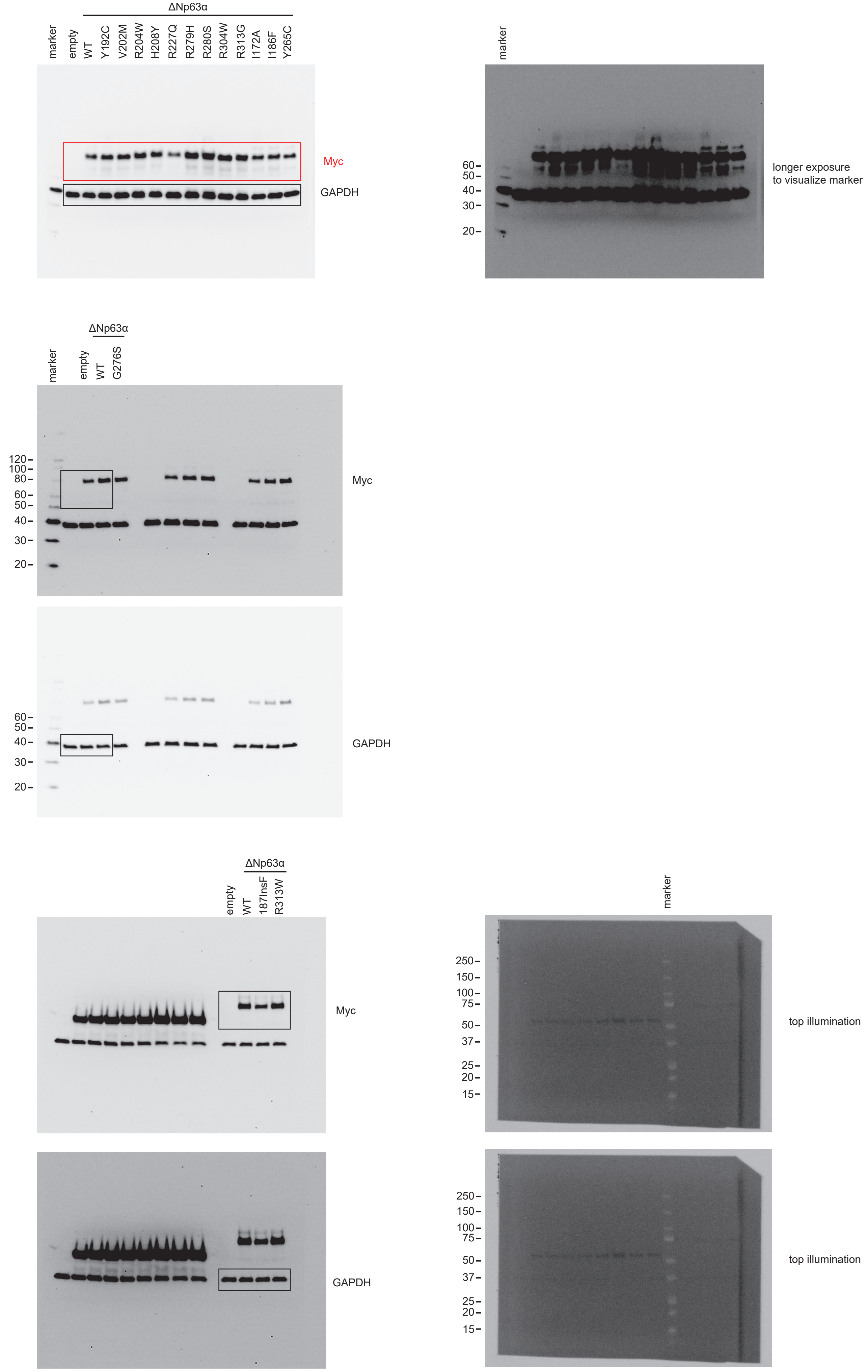
ΔNp63α
marker
empty
WT
Y192C
V202M
R204W
H208Y
R227Q
R279H
R280S
R304W
R313G
I172A
I186F
Y265C
Myc
GAPDH
marker
60
50
40
30
20
longer exposure
to visualize marker
ΔNp63α
marker
empty
WT
G276S
120
100
80
60
50
40
30
20
Myc
60
50
40
30
20
GAPDH
ΔNp63α
empty
WT
187InsF
R313W
Myc
GAPDH
marker
250
150
100
75
50
37
25
20
15
top illumination
250
150
100
75
50
37
25
20
15
top illumination

Figure S2B - continued

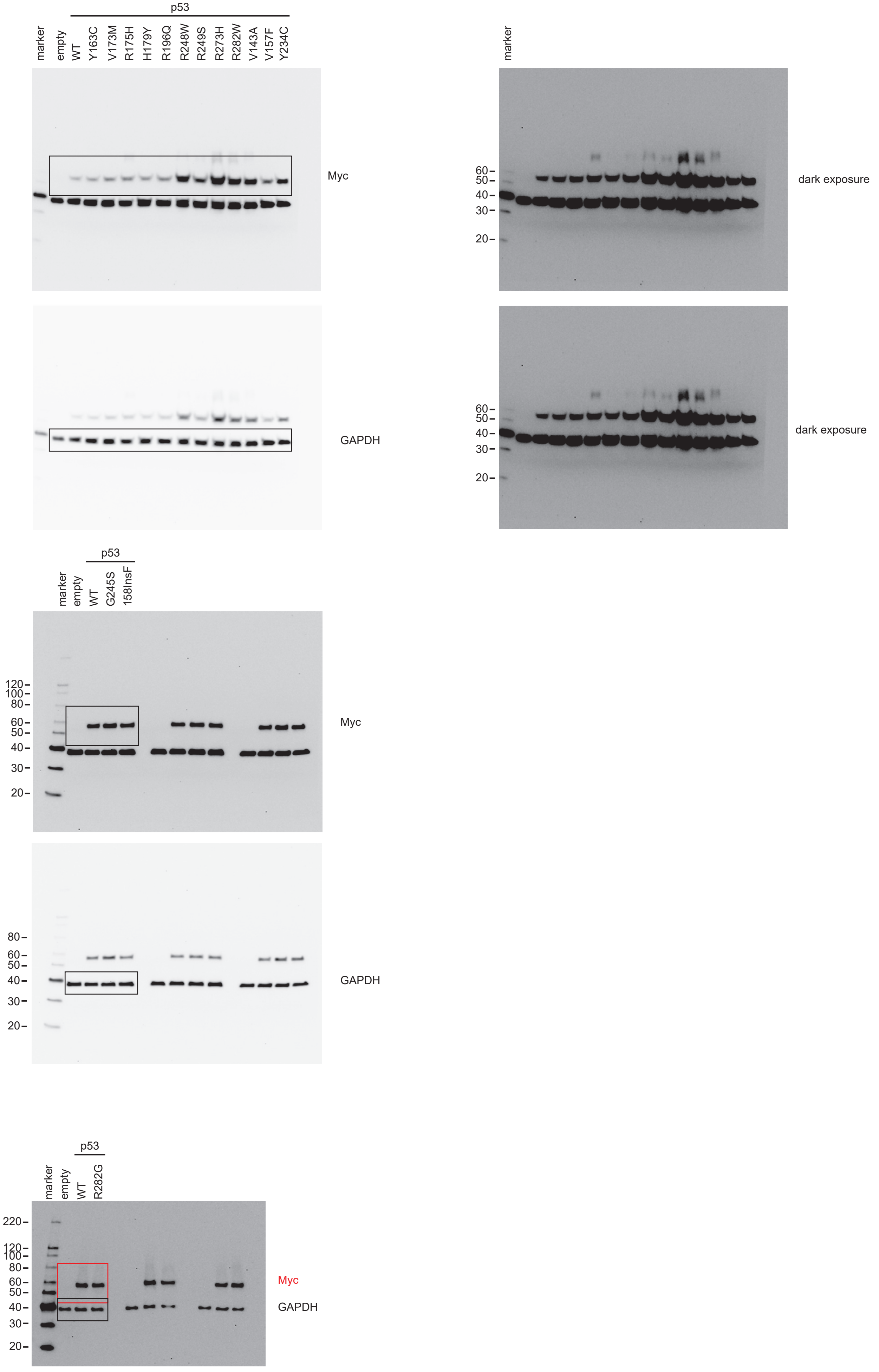

Figure S2D

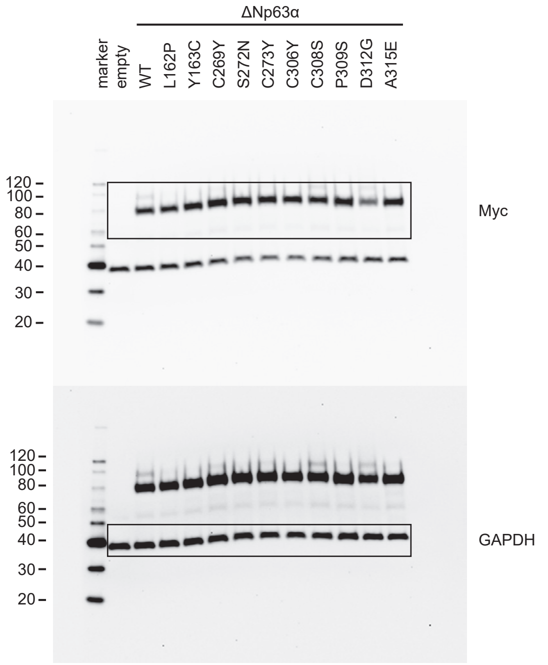

Figure S2F

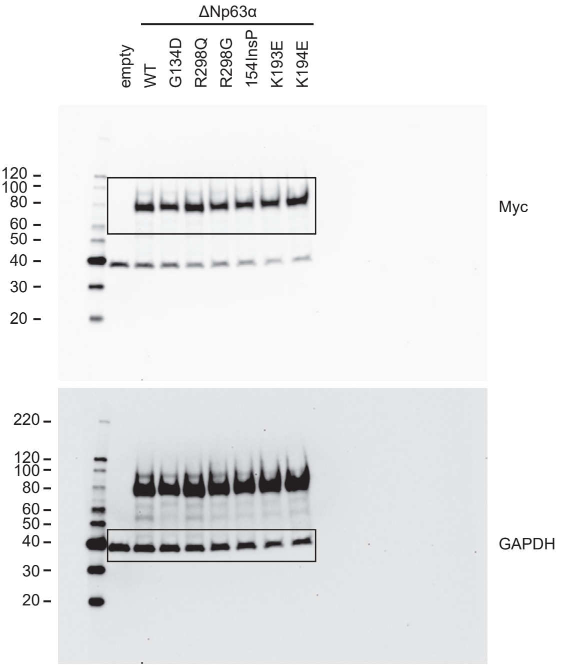

Figure S2H

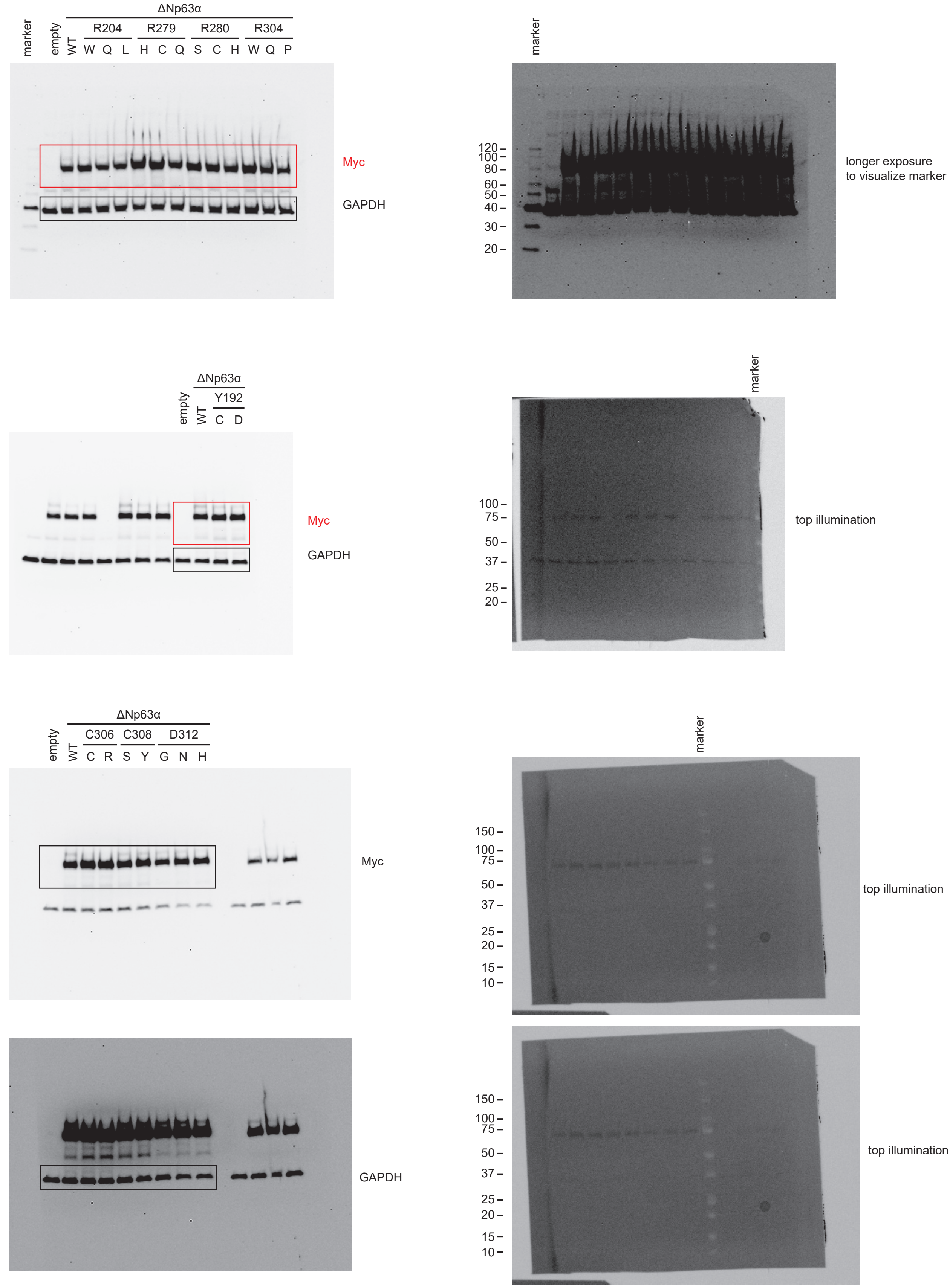
ΔNp63α
marker
empty
WT
R204
W Q L
R279
H C Q
R280
S C H
R304
W Q P
Myc
GAPDH
marker
120 –
100 –
80 –
60 –
50 –
40 –
30 –
20 –
longer exposure
to visualize marker
ΔNp63α
empty
WT
Y192
C D
Myc
GAPDH
marker
100 –
75 –
50 –
37 –
25 –
20 –
top illumination
ΔNp63α
empty
WT
C306
C R
C308
S Y
D312
G N H
Myc
marker
150 –
100 –
75 –
50 –
37 –
25 –
20 –
15 –
10 –
top illumination
GAPDH
150 –
100 –
75 –
50 –
37 –
25 –
20 –
15 –
10 –
top illumination

Figure S2J

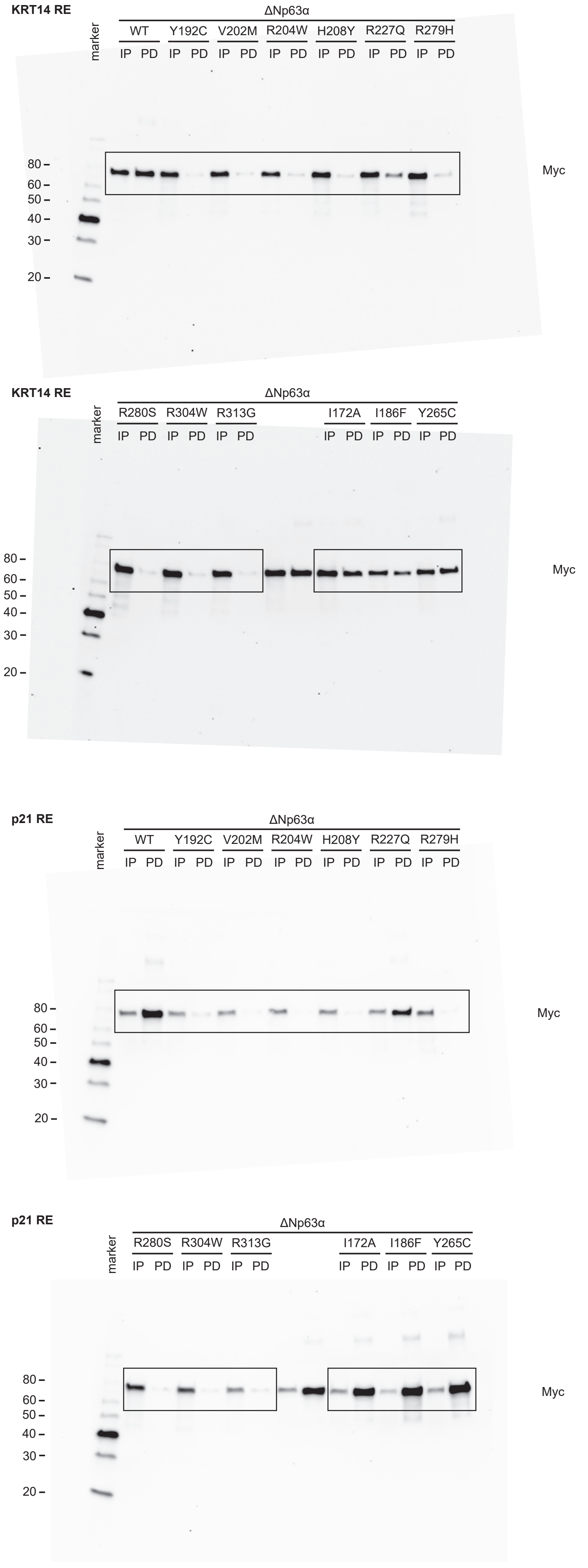

KRT14 RE
ΔNp63α
marker
WT
Y192C
V202M
R204W
H208Y
R227Q
R279H
IP
PD
80
60
50
40
30
20
Myc
KRT14 RE
ΔNp63α
marker
R280S
R304W
R313G
I172A
I186F
Y265C
IP
PD
80
60
50
40
30
20
Myc
p21 RE
ΔNp63α
marker
WT
Y192C
V202M
R204W
H208Y
R227Q
R279H
IP
PD
80
60
50
40
30
20
Myc
p21 RE
ΔNp63α
marker
R280S
R304W
R313G
I172A
I186F
Y265C
IP
PD
80
60
50
40
30
20
Myc

Figure 4B

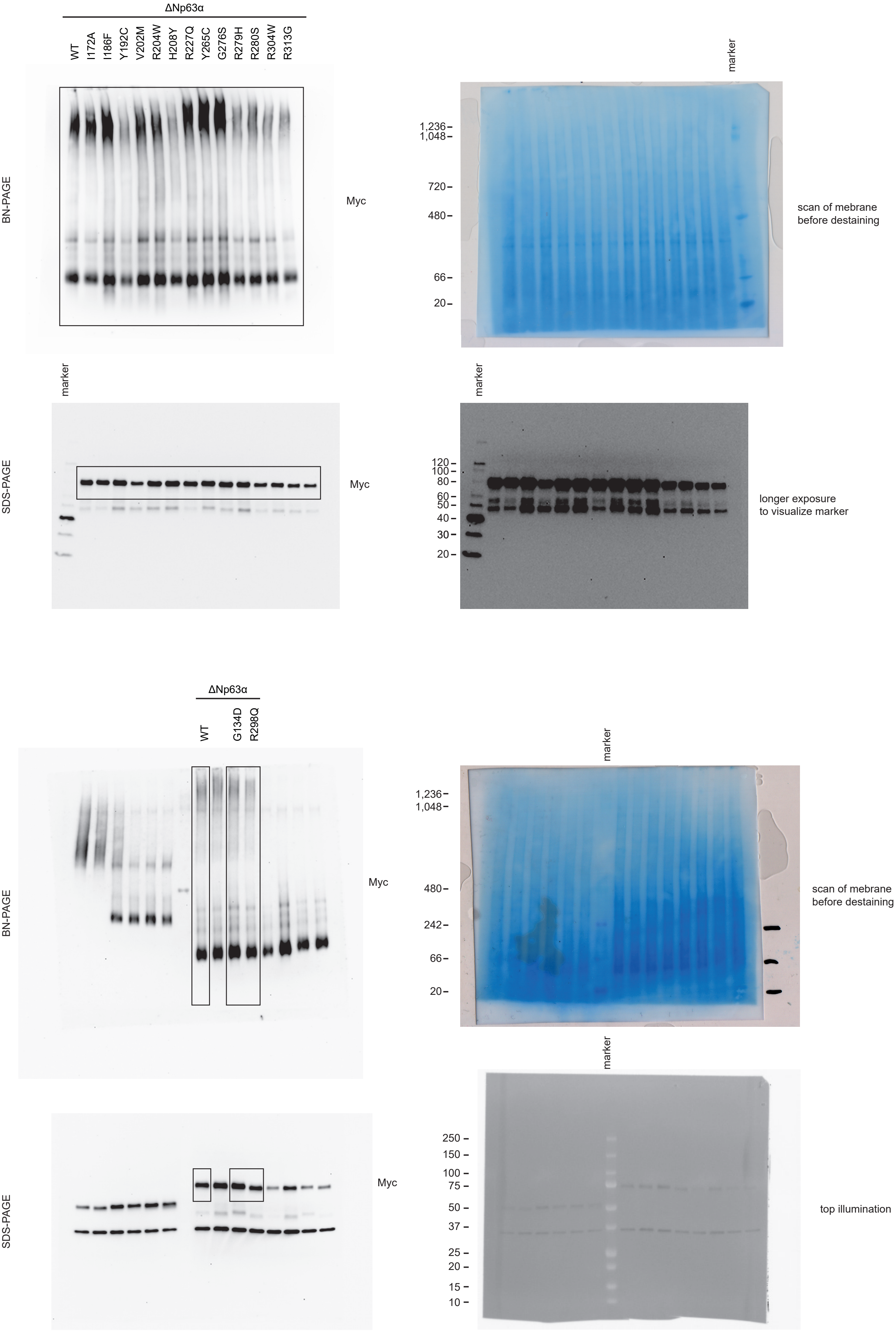
ΔNp63α
WT
I172A
I186F
Y192C
V202M
R204W
H208Y
R227Q
Y265C
G276S
R279H
R280S
R304W
R313G
BN-PAGE
Myc
marker
1,236
1,048
720
480
66
20
scan of mebrane
before destaining
SDS-PAGE
Myc
120
100
80
60
50
40
30
20
longer exposure
to visualize marker
ΔNp63α
WT
G134D
R298Q
BN-PAGE
Myc
marker
1,236
1,048
480
242
66
20
scan of mebrane
before destaining
SDS-PAGE
Myc
marker
250
150
100
75
50
37
25
20
15
10
top illumination

Figure 4B - continued

BN-PAGE

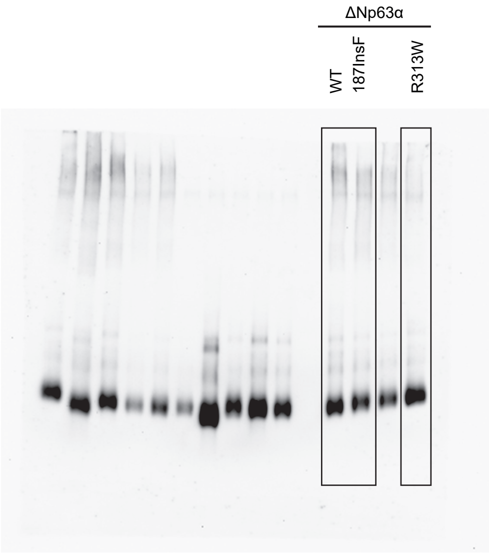

Myc

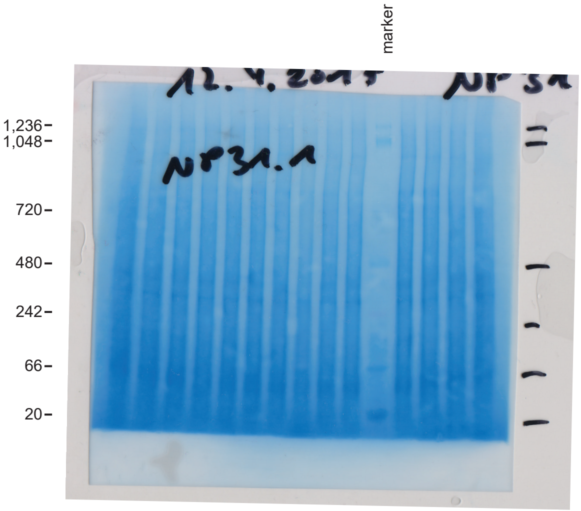

scan of mebrane before destaining

SDS-PAGE

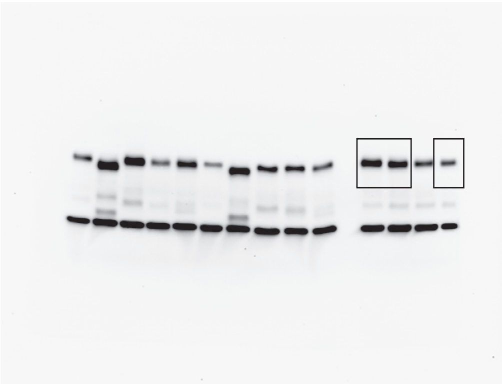

Myc

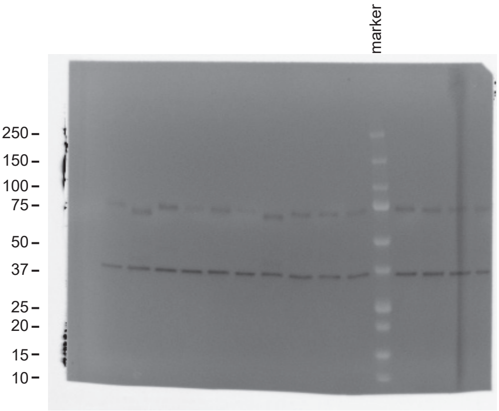

top illumination

Figure S4B

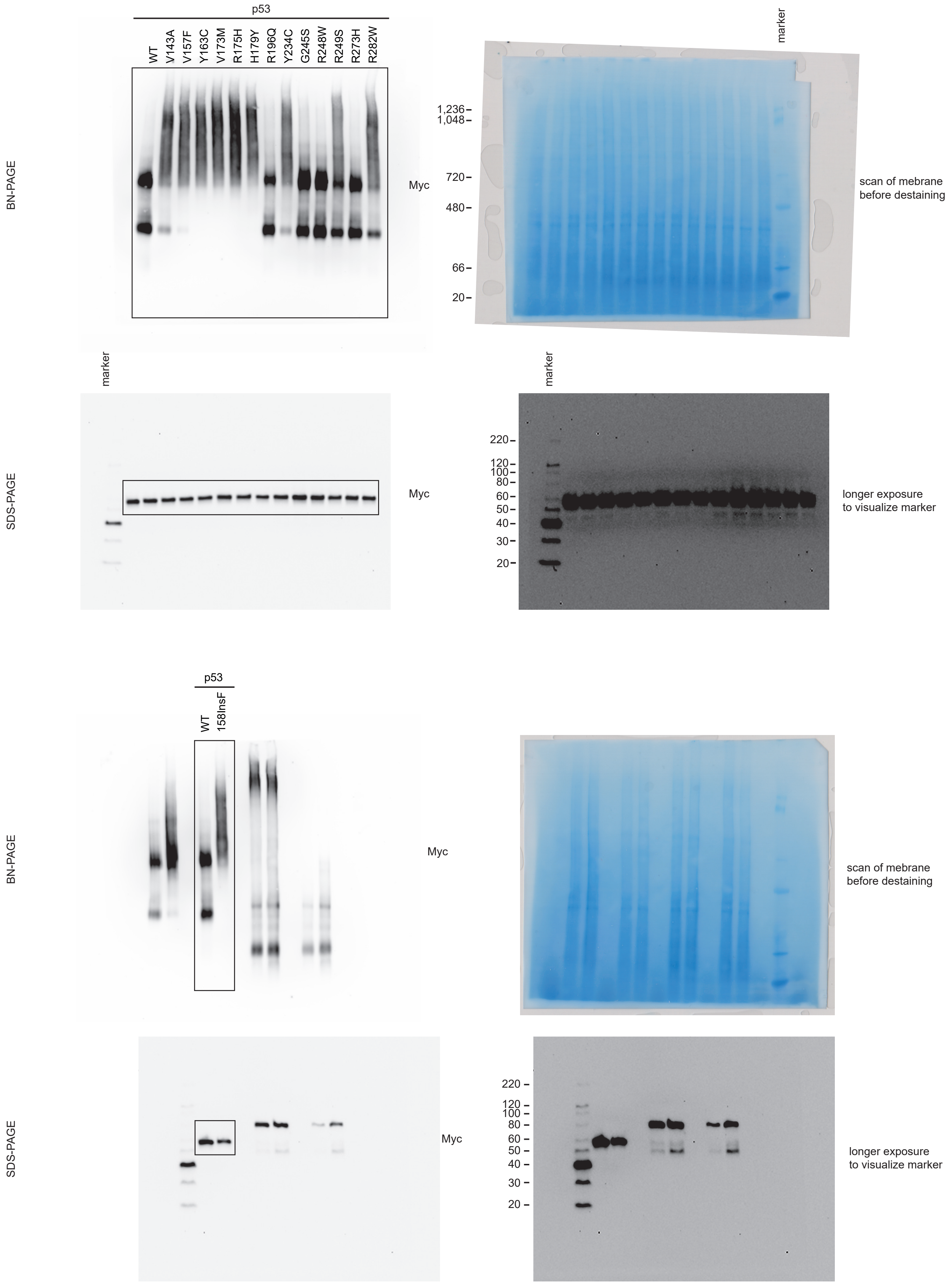
p53
WT
V143A
V157F
Y163C
V173M
R175H
H179Y
R196Q
Y234C
G245S
R248W
R249S
R273H
R282W
BN-PAGE
Myc
marker
1,236
1,048
720
480
66
20
scan of mebrane
before destaining
SDS-PAGE
marker
Myc
marker
220
120
100
80
60
50
40
30
20
longer exposure
to visualize marker
p53
WT
158InsF
BN-PAGE
Myc
scan of mebrane
before destaining
SDS-PAGE
Myc
220
120
100
80
60
50
40
30
20
longer exposure
to visualize marker

Figure S4C

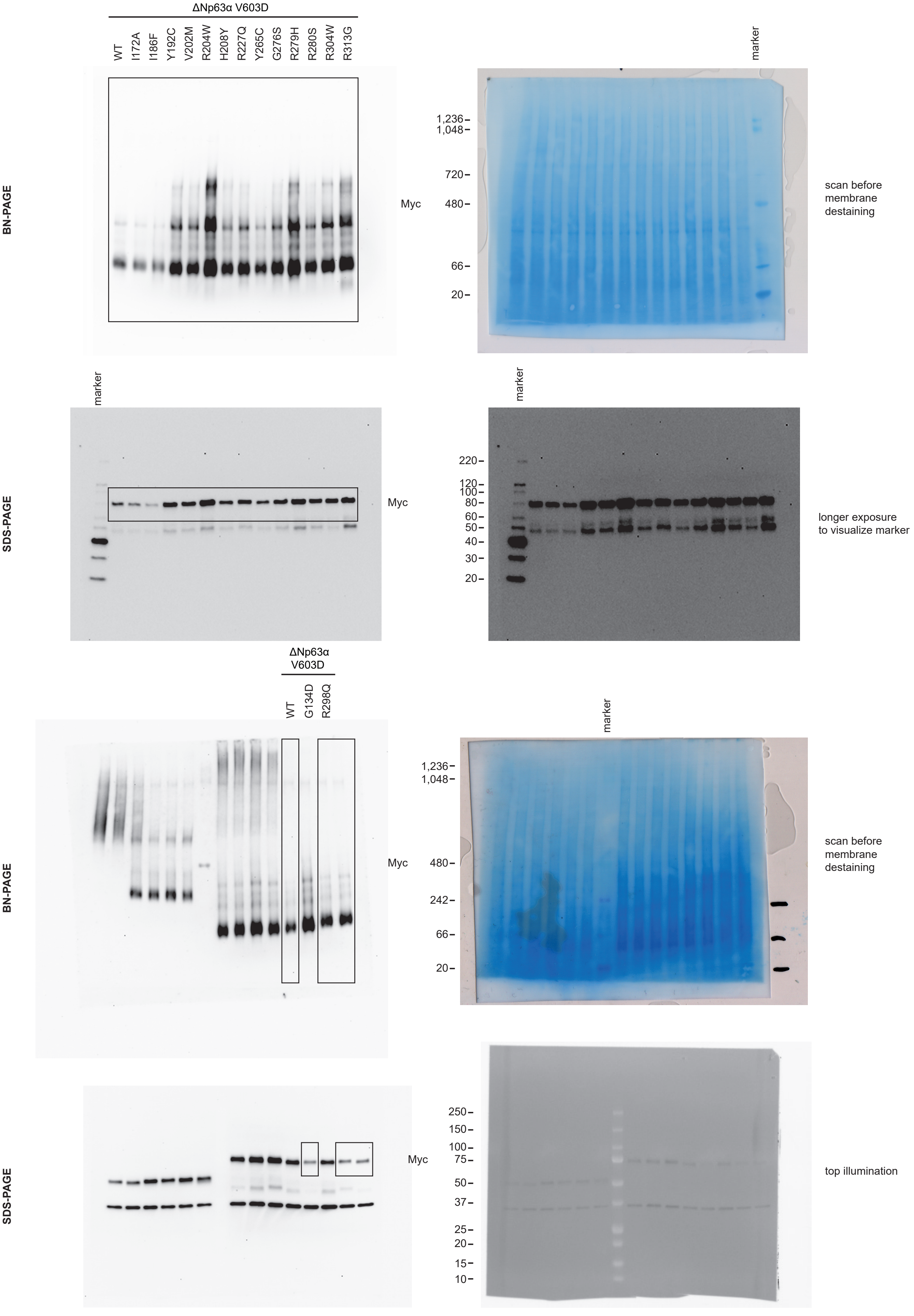

ΔNp63α V603D
WT
I172A
I186F
Y192C
V202M
R204W
H208Y
R227Q
Y265C
G276S
R279H
R280S
R304W
R313G
marker
BN-PAGE
Myc
1,236
1,048
720
480
66
20
scan before membrane destaining
marker
SDS-PAGE
Myc
marker
220
120
100
80
60
50
40
30
20
longer exposure to visualize marker
ΔNp63α V603D
WT
G134D
R298Q
marker
BN-PAGE
Myc
1,236
1,048
480
242
66
20
scan before membrane destaining
SDS-PAGE
Myc
250
150
100
75
50
37
25
20
15
10
top illumination

Figure S4C - continued

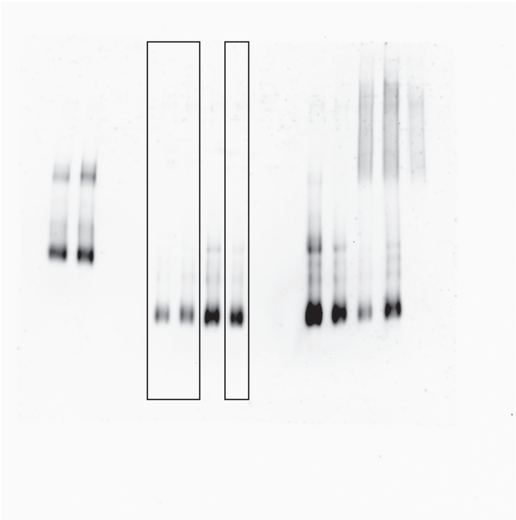

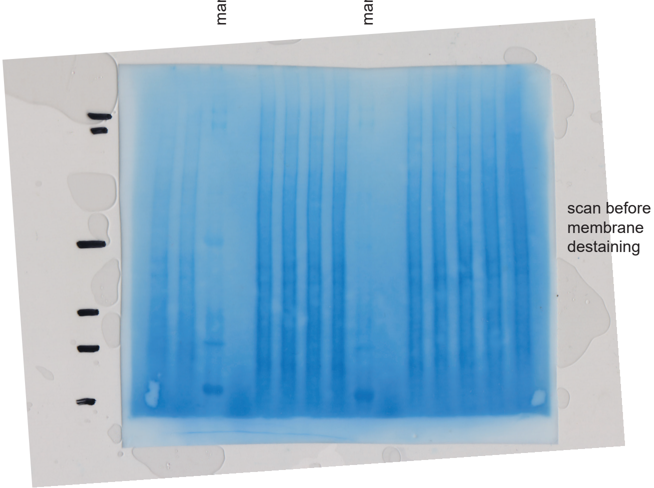

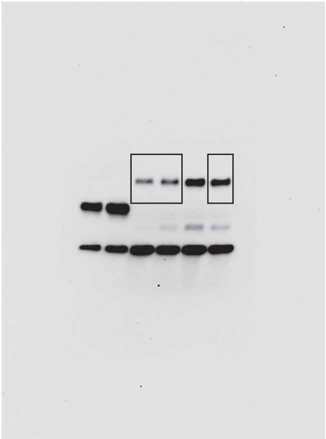

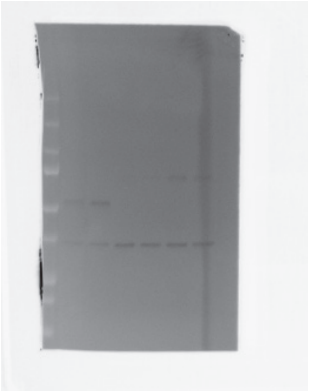

Figure S4E

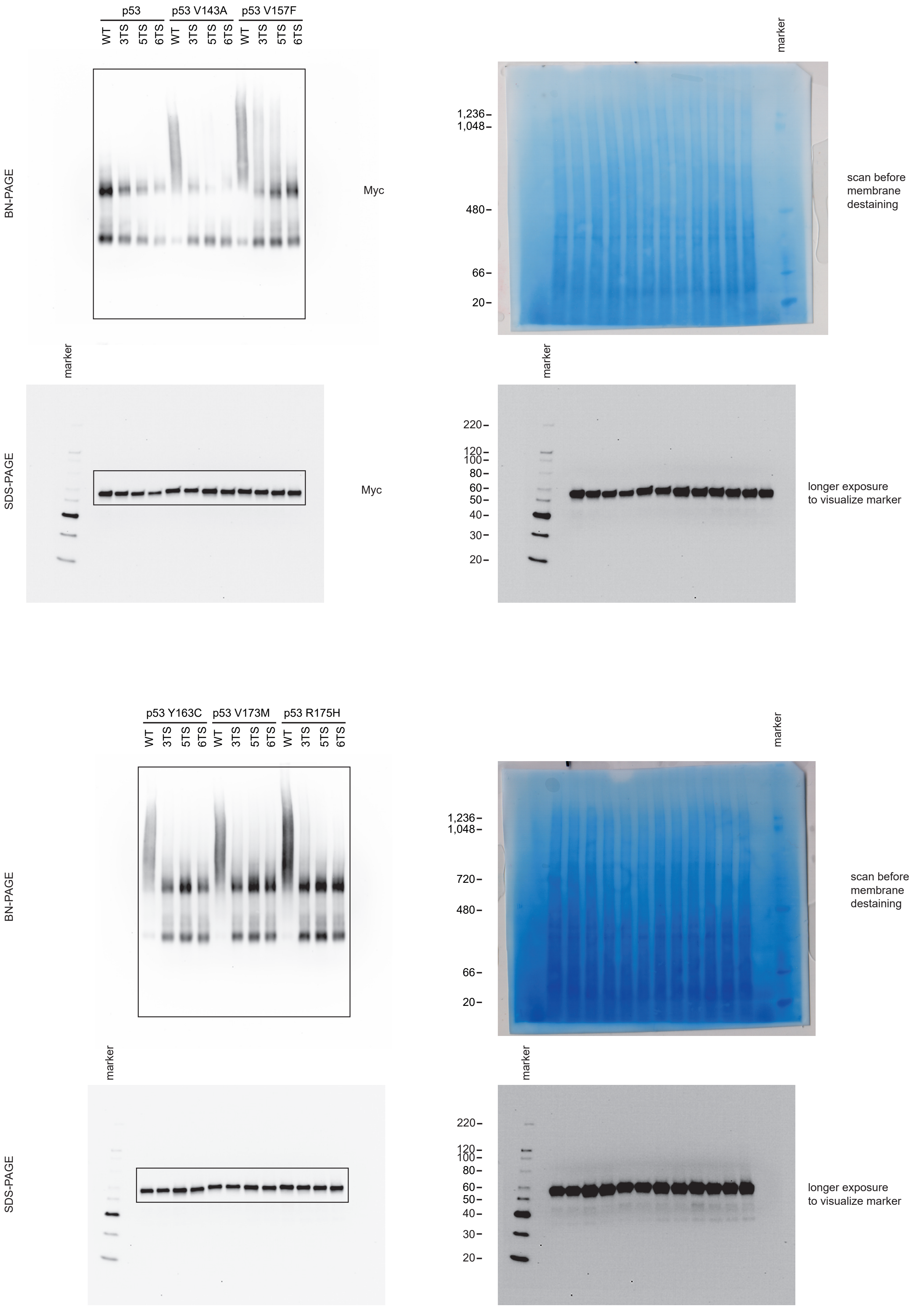

p53
p53 V143A
p53 V157F
WT
3TS
5TS
6TS
marker
BN-PAGE
Myc
1,236
1,048
480
66
20
scan before membrane destaining
SDS-PAGE
220
120
100
80
60
50
40
30
longer exposure to visualize marker
p53 Y163C
p53 V173M
p53 R175H
720

Figure S4E - continued

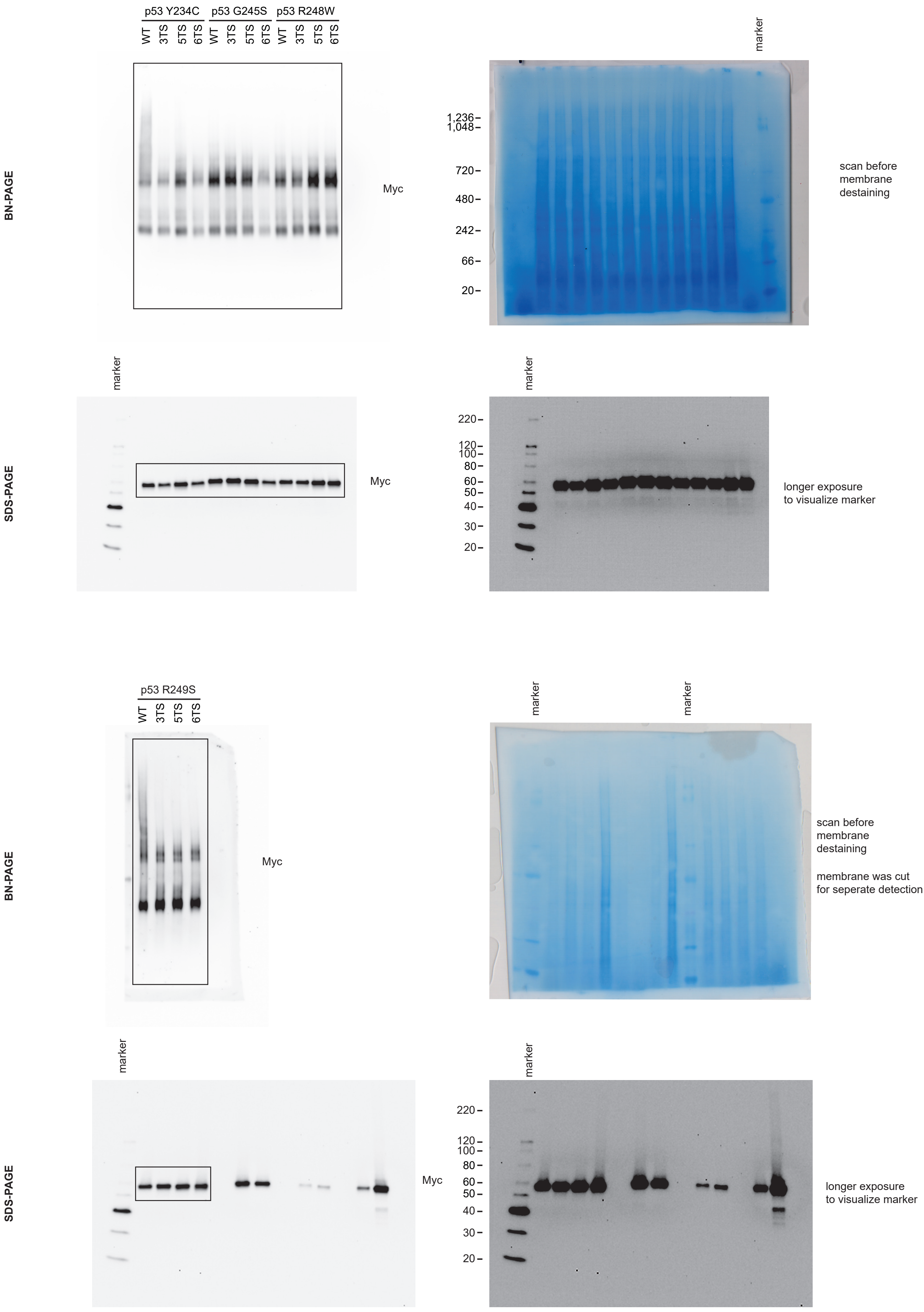

Figure S4E - continued

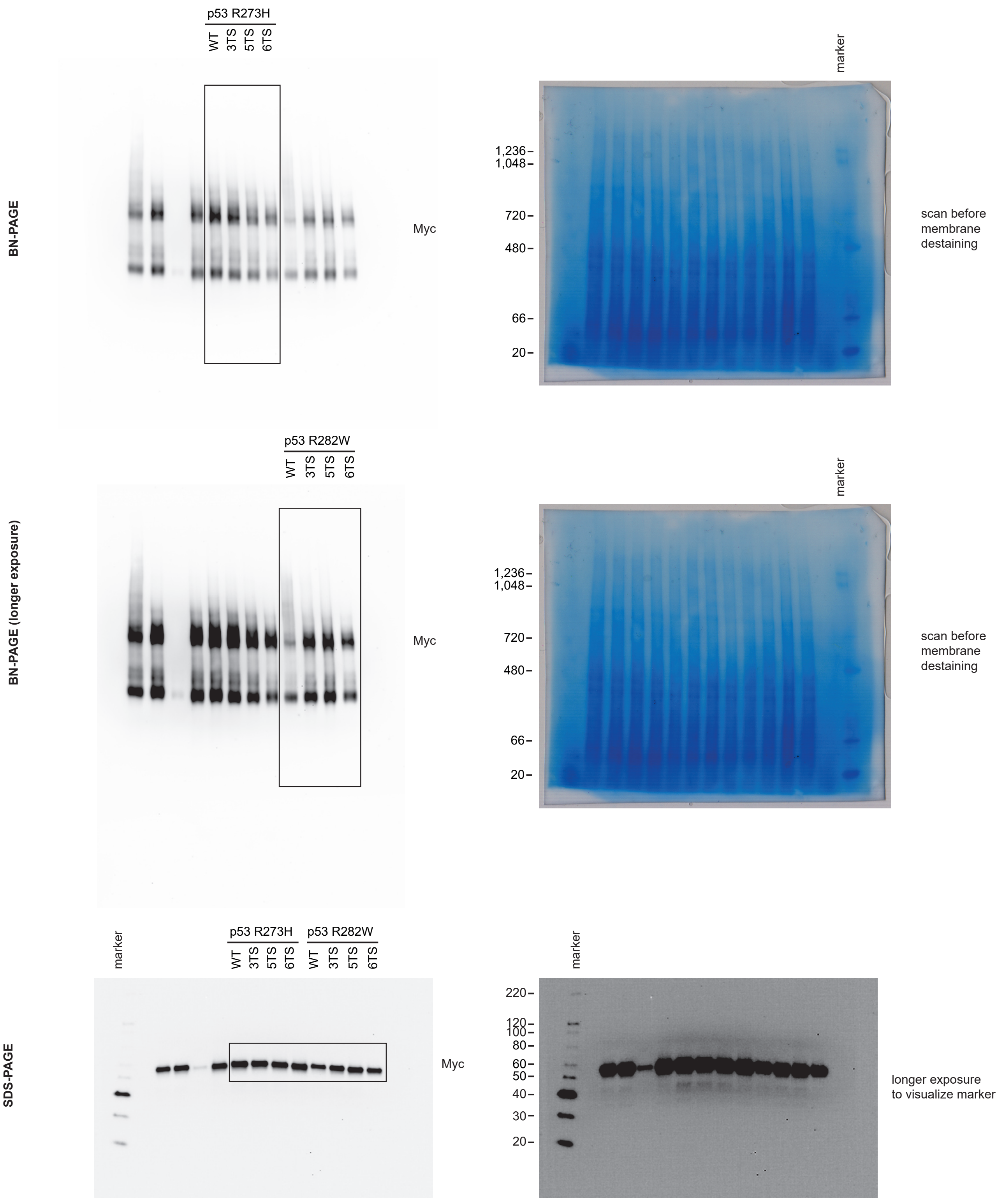

Figure S4E - continued

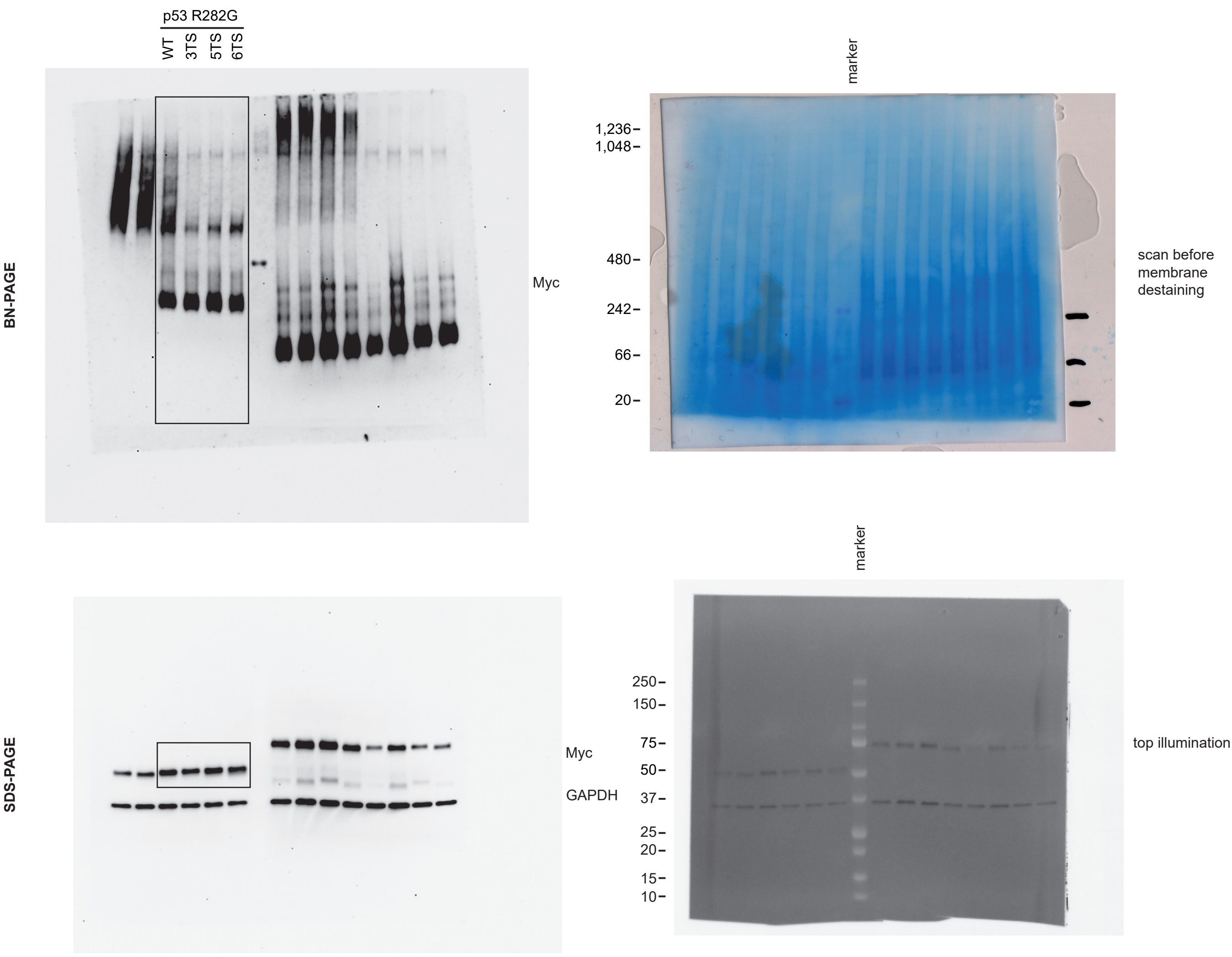

Figure S4G

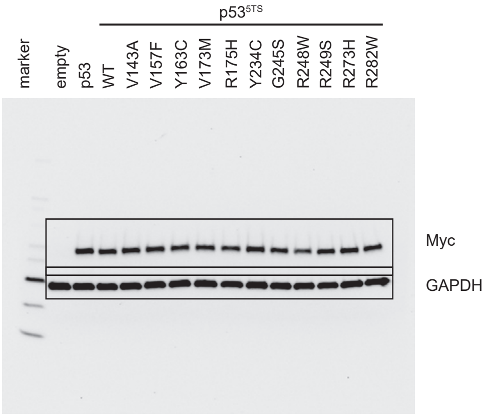
p53[5TS]
marker
empty
p53
WT
V143A
V157F
Y163C
V173M
R175H
Y234C
G245S
R248W
R249S
R273H
R282W
Myc
GAPDH

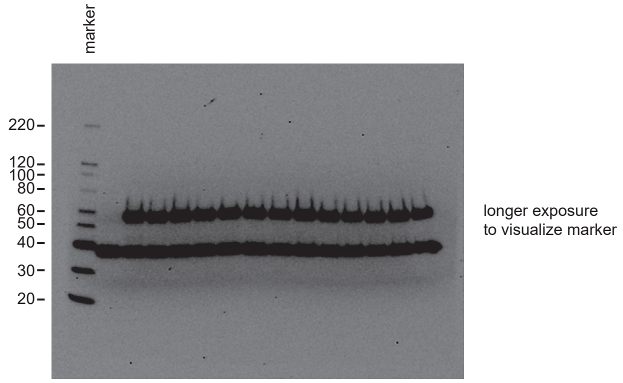
marker
220
120
100
80
60
50
40
30
20
longer exposure
to visualize marker

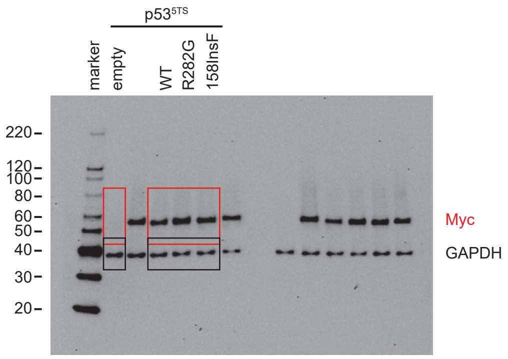
p53[5TS]
marker
empty
WT
R282G
158InsF
220
120
100
80
60
50
40
30
20
Myc
GAPDH

Figure 6C

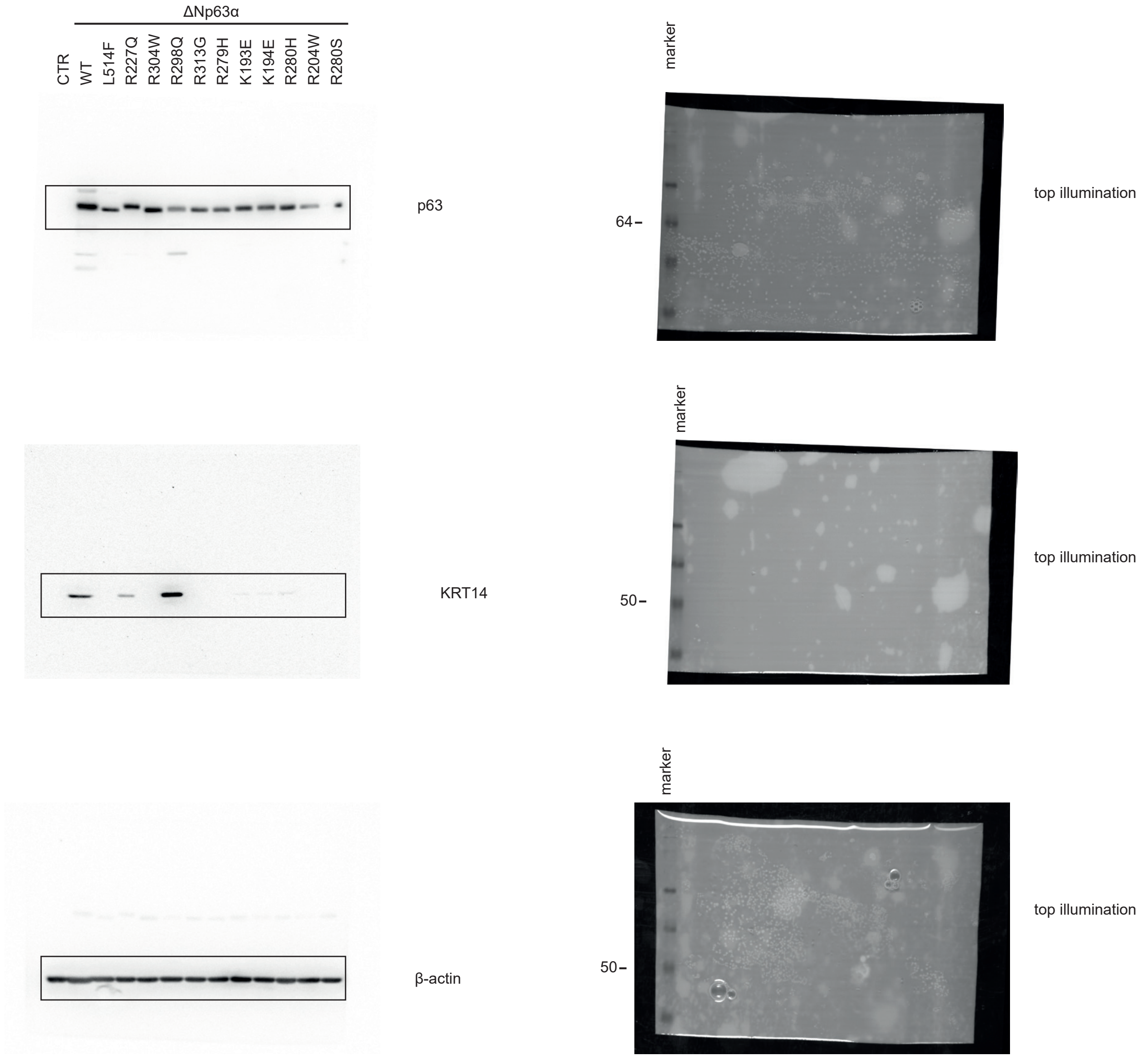

ΔNp63α
CTR
WT
L514F
R227Q
R304W
R298Q
R313G
R279H
K193E
K194E
R280H
R204W
R280S
marker
p63
64
top illumination
marker
KRT14
50
top illumination
marker
β-actin
50
top illumination

Figure S7A

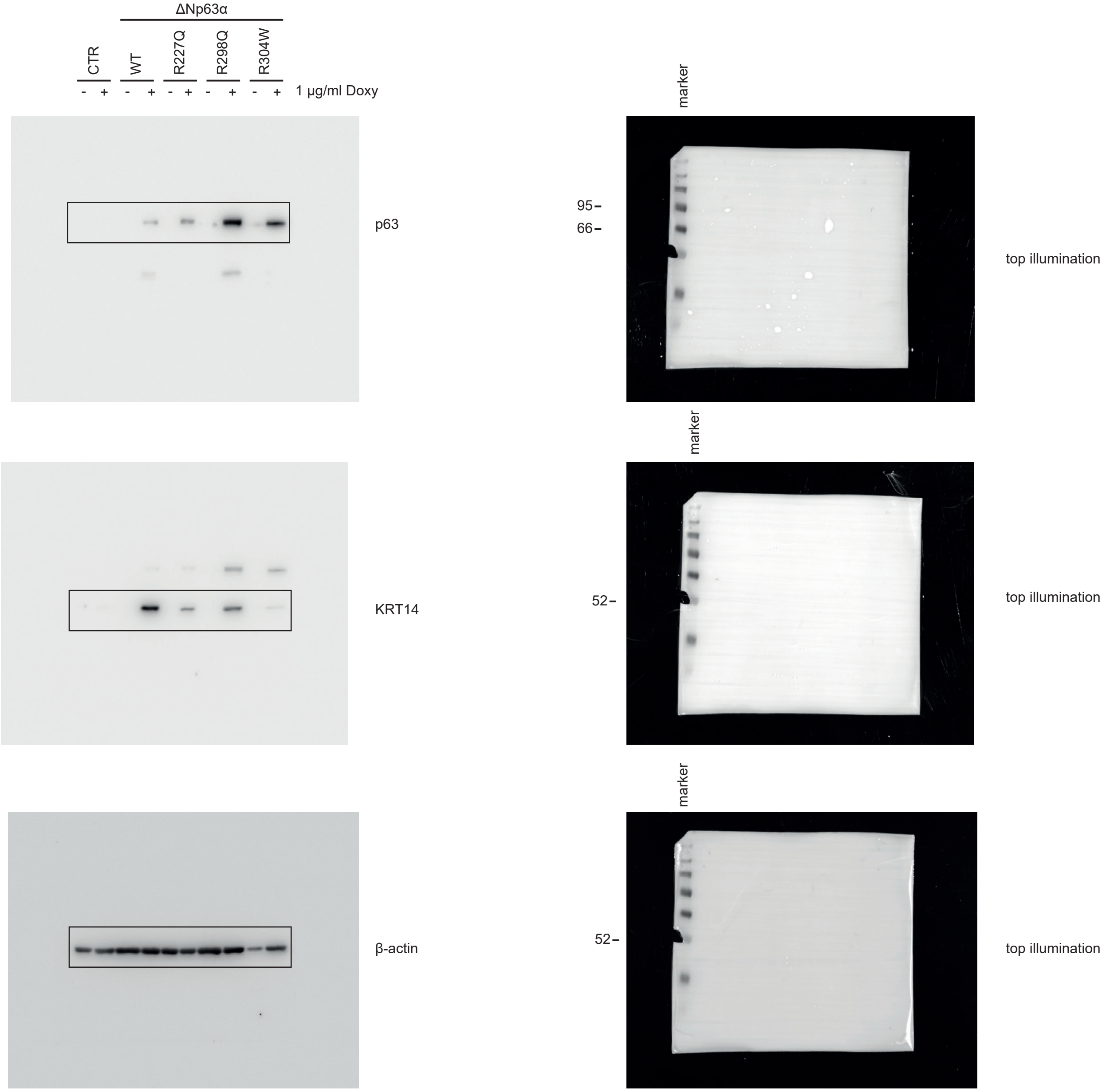
ΔNp63α
CTR
WT
R227Q
R298Q
R304W
- + - + - + - + - +
1 µg/ml Doxy
marker
p63
95
66
top illumination
KRT14
52
β-actin
