# Supplementary material for: Disease-related p63 DBD mutations impair DNA binding by distinct mechanisms and varying degree
Source: Cell Death Dis. 2023 Apr 18;14(4):274. doi: 10.1038/s41419-023-05796-y (PMC10113246; doi:10.1038/s41419-023-05796-y)
Supplement: Supplementary file 11 — Supplementary Table S2 [file 41419_2023_5796_MOESM11_ESM.docx]

| **p63** | | | **p53** | | | |
| --- | --- | --- | --- | --- | --- | --- |
| **aa** | **Syndrome mutations*** | **Hotspot?** | **aa** | **Cancer mutations*^,#^** | **Hotspot?** | **Classification^‡^** |
| P127 | L |  | P98 | (S/L/T) |  |  |
| S128 | F |  | S99 | (F/P) |  |  |
| G134 | D/V |  | G105 | (C/V/D/R/S/A) |  |  |
| W153 | R |  | C124 | (X/R/G/Y/F/S) |  |  |
| K161 | E |  | K132 | (N/R/E/M/Q/T/X) |  |  |
| L162 | P |  | M133 | (K/R/T/I/L/V) |  |  |
| Y163 | C/D |  | F134 | (L/C/V/S/I) |  |  |
| I172 |  |  | V143 | (M/A/E/G/L) |  | core, temperature sensitive |
| V185 | I |  | R156 | (P/H/C/G/L/S) |  |  |
| I186 |  |  | V157 | F (G/D/I/L/A) |  | core, temperature sensitive |
| Y192 | C/D |  | Y163 | C (N/H/X/D/S) |  |  |
| K193 | E |  | K164 | (E/X/N/M/Q/T/R) |  |  |
| K194 | E |  | Q165 | (X/H/L/R/P/E) |  |  |
| V202 | M |  | V173 | (L/M/A/G/E) |  |  |
| R204 | W/Q/L | yes | R175 | H (G/L/C/S/P) | yes | indirect zinc finger |
| H208 | R/D/L/Y |  | H179 | R/Y (L/Q/N/D/P) | yes | direct zinc finger |
| R227 | Q/P | yes | R196 | X (P/Q/L/G) |  |  |
| E229 | K |  | E198 | (X/K/Q/G) |  |  |
| Y251 |  |  | Y220 | C (N/S/H/X/D) | yes | core, temperature sensitive |
| Y265 |  |  | Y234 | C (H/N/X/D/S/F) |  | core, temperature sensitive |
| C269 | Y |  | C238 | Y (F/S/R/G/W/X) |  | direct zinc finger |
| S271 | T |  | S240 | (G/R/I/C/T) |  |  |
| S272 | N/T |  | S241 | F (C/Y/A/P/T) |  |  |
| C273 | Y |  | C242 | (F/Y/S/R/G/W/X) |  | direct zinc finger |
| G275 | E |  | G244 | (C/D/S/V/A/R) |  |  |
| G276 |  |  | G245 | S/D (V/C/R/A) | yes | indirect DNA contact, cooperativity |
| R279 | H/C/S/Q | yes | R248 | Q/W (L/G/P) | yes | direct DNA contact |
| R280 | C/H/S | yes | R249 | S (M/W/G/T/K) | yes | indirect DNA contact |
| Q294 | X |  | N263 | (D/I/H/K) |  |  |
| R298 | Q/G/L | yes | R267 | (W/P/Q/L/G) |  |  |
| R304 | Q/W/G/P | yes | R273 | H/C/L (P/S/G) | yes | direct DNA contact |
| C306 | Y/R |  | C275 | (Y/F/R/W/G/S/X) |  |  |
| A307 | D/G |  | A276 | (P/D/V/S/T/G) |  |  |
| C308 | Y/S |  | C277 | (F/Y/G/X/W/R/S) |  |  |
| P309 | S |  | P278 | (S/L/R/T/A/H) |  |  |
| G310 | E |  | G279 | (E/R/V/W) |  |  |
| R311 | G |  | R280 | (T/K/G/I/S/X) |  |  |
| D312 | N/E/G/H |  | D281 | (H/E/N/G/Y/V/A) |  |  |
| R313 | G |  | R282 | W (G/Q/P/L) | yes | temperature sensitive |
| A315 | E |  | T284 | (P/A/I/S) |  |  |
| D316 | H |  | E285 | K (X/V/Q/G/A) |  |  |

*****: p63 and p53 mutations resulting from a CpG site are underscored

**^#^**: p53 mutation with a frequency below 0.5% are parenthesized (Supplementary Table 1).

**^‡^**: Classification of the p53 mutations is stated according to Joerger et al. (2007).
